# Investigation of the Genetic Diversity and Quantitative Trait Loci Accounting for Important Agronomic and Seed Quality Traits in *Brassica carinata*

**DOI:** 10.3389/fpls.2017.00615

**Published:** 2017-04-24

**Authors:** Wenshan Zhang, Dandan Hu, Rosy Raman, Shaomin Guo, Zili Wei, Xueqi Shen, Jinling Meng, Harsh Raman, Jun Zou

**Affiliations:** ^1^National Key Laboratory of Crop Genetic Improvement, Key Laboratory of Rapeseed Genetic Improvement, Ministry of Agriculture China, Huazhong Agricultural UniversityWuhan, China; ^2^Graham Centre for Agricultural Innovation (an Alliance between the Charles Sturt University and NSW Department of Primary Industries), Wagga Wagga Agricultural InstituteWagga Wagga, NSW, Australia

**Keywords:** genetic diversity, quantitative trait loci, *Brassica carinata*, agronomic traits, seed quality, subgenome

## Abstract

*Brassica carinata* (BBCC) is an allotetraploid in *Brassicas* with unique alleles for agronomic traits and has huge potential as source for biodiesel production. To investigate the genome-wide molecular diversity, population structure and linkage disequilibrium (LD) pattern in this species, we genotyped a panel of 81 accessions of *B. carinata* with genotyping by sequencing approach DArTseq, generating a total of 54,510 polymorphic markers. Two subpopulations were exhibited in the *B. carinata* accessions. The average distance of LD decay (*r*^2^ = 0.1) in B subgenome (0.25 Mb) was shorter than that of C subgenome (0.40 Mb). Genome-wide association analysis (GWAS) identified a total of seven markers significantly associated with five seed quality traits in two experiments. To further identify the quantitative trait loci (QTL) for important agronomic and seed quality traits, we phenotyped a doubled haploid (DH) mapping population derived from the “YW” cross between two parents (Y-BcDH64 and W-BcDH76) representing from the two subpopulations. The YW DH population and its parents were grown in three contrasting environments; spring (Hezheng and Xining, China), semi-winter (Wuhan, China), and spring (Wagga Wagga, Australia) across 5 years for QTL mapping. Genetic bases of phenotypic variation in seed yield and its seven related traits, and six seed quality traits were determined. A total of 282 consensus QTL accounting for these traits were identified including nine major QTL for flowering time, oleic acid, linolenic acid, pod number of main inflorescence, and seed weight. Of these, 109 and 134 QTL were specific to spring and semi-winter environment, respectively, while 39 consensus QTL were identified in both contrasting environments. Two QTL identified for linolenic acid (B3) and erucic acid (C7) were validated in the diverse lines used for GWAS. A total of 25 QTL accounting for flowering time, erucic acid, and oleic acid were aligned to the homologous QTL or candidate gene regions in the C genome of *B. napus*. These results would not only provide insights for genetic improvement of this species, but will also identify useful genetic variation hidden in the C^c^ subgenome of *B. carinata* to improve canola cultivars.

## Introduction

Ethiopian mustard (*Brassica carinata* A. Braun 2*n* = 4*x* = 36, genomes BBCC) is an important member of the family Brassicaceae and is commercially grown for edible vegetable oil, and as vegetable crop in Ethiopia. This crop is also being exploited for biodiesel as a source of a renewable energy (Warwick et al., 2006). It consists of the two homologous genomes, B and C, and may have originated as an allotetraploid species as a result of spontaneous hybridization between diploid species; *Brassica nigra* (2*n* = 2*x* = 14, genome BB) and *Brassica oleracea* (2*n* = 2*x* = 18, genome CC) in Ethiopia (Nagaharu, 1935; Lukens et al., 2004; Warwick, 2011). *B. carinata* harbors useful genes for resistance to abiotic and biotic stresses (Getinet et al., 1996), and therefore has been used as a donor for introgression of genes to improve and widen the gene pool of *Brassica rapa, Brassica napus*, and *Brassica juncea* germplasm (Meng et al., 1998; Xiao et al., 2010; Wei et al., 2016).

Relative to its other close relatives, *B. rapa, B. napus*, and *B. juncea*, genetic improvement of this crop has been limited due to lower grain yield, “poor” canola oil quality attributes and unavailability of genetic tools and resources. So far, the genomes of *B. rapa, B. oleracea, B. napus, B. nigra*, and *B. juncea* have been sequenced and published (Wang et al., 2011; Chalhoub et al., 2014; Liu et al., 2014; Parkin et al., 2014; Yang et al., 2016), while the genome of *B. carinata* is sequenced but has not been published (Parkin, per comm.).

Research has shown that a limited genetic variation exists in *B. carinata* (Jiang et al., 2007; Guo et al., 2012). In order to increase the rate of genetic gains in breeding programs, QTL (quantitative trait loci) mapping has been successfully used to identify both qualitative and quantitative loci in several crops including vegetable and oilseed brassicas. QTL for various agronomic traits such as grain yield and its components, flowering time, seed quality, and tolerance to biotic and abiotic stresses have been identified in *B. rapa* (Rahman et al., 2014; Hirani et al., 2016), *B. juncea* (Singh et al., 2012; Bagheri et al., 2013; Dhaka et al., 2016) and *B. napus* (Snowdon and Friedt, 2004; Shi et al., 2009; Zou et al., 2010; Yong et al., 2015; Li et al., 2016; Raman et al., 2016). However, reports on such analyses in *B. carinata* has been very limited. A genetic linkage map of the doubled haploid (DH) population (named as YW population) derived from a cross between two DH accessions of *B. carinata*, Y-BcDH64 (yellow petal) and W-BcDH76 (white petal), was constructed using a total of 212 amplified fragment length polymorphism (AFLP), intron based polymorphism (IBP), sequence-related amplified polymorphism (SRAP), and simple sequence repeats (SSR) markers, and covered 1,703 cM with a marker density of 8 cM between adjacent loci (Guo et al., 2012). In addition, a high density genetic linkage map of the YW DH population integrating existing 212 markers (Guo et al., 2012) and 3,819 presence-absence markers based on genotyping-by-sequencing, DArTseq markers (the traditional DArT and next-generation sequencing technique called DArTseq, Raman et al., 2014), was established with increasing genome-wide coverage (2,048 cM) (Zou et al., 2014). The genetic map of YW DH population was subsequently used for the QTL mapping of genetic loci involved in petal color, another tip color, seed coat color (Guo et al., 2012), and flowering and budding time (Zou et al., 2014). However, a limited phenotypic datasets especially on agronomic traits were available for QTL detection and routine marker-assisted selection.

In the present study, we genotyped a set of 81 *B. carinata* accessions to investigate the genetic diversity, population structure and the linkage disequilibrium (LD) pattern of *B. carinata* using DArTseq markers. We also conducted genome-wide association study (GWAS) to detect associations between markers and six seed quality traits among the 81 *B. carinata* accessions evaluated in two experiments. We further phenotyped the YW DH population for 14 seed yield, yield-related traits, and oil quality traits across different agro-climatic conditions for QTL mapping, and compared those results with the putative candidate genes or QTL identified in the C genome of *B. napus*, as well as the associated markers accounting for genotypic variation in seed quality traits of the diverse lines of *B. carinata*.

## Materials and methods

### Plant materials

DH mapping population (185 lines) of *B. carinata* was developed from an F_1_ cross between the DH parental lines, Y-BcDH64 and W-BcDH76 (Guo et al., 2012). In addition, a set of 81 diverse accessions (Supplementary Table 1; Jiang et al., 2007) of *B. carinata* including the parents of the YW DH population, mainly of Ethiopian origin, collected from Centre for Genetic Resources, Wageningen, The Netherlands and Germany, were used for genetic diversity analyses. All accessions were selfed to avoid any cross pollination.

### DNA extraction, genotyping, and the physical position of the markers

DNA from the *B. carinata* accessions was extracted from bulked young leaf tissue using the DNA extraction Kit (DNeasy Plant Mini Kit, QIAGEN) and sent to DArT P/L (www.diversityarrays.com) for DArTseq based genotyping as described previously (Raman et al., 2014). Both presence-absence markers and SNP markers, collectively called “DArTseq markers,” were scored by DArT P/L.

The 69-bp long sequence of the filtered DArTseq markers identified in 81 *B. carinata* accessions were aligned to the reference genome of *B. nigra* (Yang et al., 2016), *B. napus* (Chalhoub et al., 2014), and *B. oleracea* (Parkin et al., 2014) by Blast using an *e*-value threshold of *e*^−10^ (Altschul et al., 1990) with ≥60 bp match length. The top blast hits for the sequences were assigned as the physical position of the corresponding markers. The blast matches to multiple loci with the same top *e*-value were considered with multiple positions, and assigned to the class of unassigned markers without unique positions to the reference genome.

In order to compare the QTL detected in the C genome of YW DH population with the QTL or candidate gene identified in *B. napus*, the physical position of the markers mapping to the C genome of YW DH population were assigned according to the top blast hits for the sequences of each marker against the reference C genome of *B. napus* (Chalhoub et al., 2014) by Blast using an *e*-value threshold of *e*^−10^ (Altschul et al., 1990) with ≥62 bp match length. If the blast matches to multiple loci had the same top *e*-value, the locus which showed the same chromosomal localization of the marker as in the genetic and physical map, was considered as the right location of the marker.

### Population structure, genetic relatedness, and linkage disequilibrium analysis

The population structure was estimated with software Structure 2.3.4 using Bayesian clustering and the admixture model (Pritchard et al., 2000). The number of subgroups (*K*) was set from 1 to 10 with three independent runs. The optimum number of subpopulations was determined by log likelihood of the data [LnP(D)] and Δ*K* method described by Evanno et al. (2005). The Q matrix was assigned to a sub-population (Remington et al., 2001). Nei's genetic distance (Nei et al., 1983) was calculated and used for the unrooted phylogeny reconstruction using a neighbor-joining (NJ) method as implemented in Powermaker version 3.25 (Liu and Muse, 2005). Phylogenetic tree was viewed using MEGA 4.0 (Tamura et al., 2007). The markers with unique position were used to estimate the LD of *B. carinata*. The parameter *r*^2^ (the squared Pearson correlation coefficient) between all pairs of SNP markers was used to estimate LD by the software package TASSEL 5.0 (Bradbury et al., 2007).

### Genome-wide association analysis

The genome-wide association analysis with four statistical models was performed by TASSEL version 5.0 (Bradbury et al., 2007). The four models are as follows: the general linear model (GLM) included a naïve model without controlling for population structure; the Q model which controlled for population structure; the mixed linear model (MLM) including kinship and the Q+K model which controlled for both population structure and kinship. The threshold for the significance of associations between markers and traits, *P* < 8.32 × 10^−5^ (*P* = 1/total markers used; −log_10_ (1/12,030) = 4.08) as in Li et al. (2016) was used in this study.

### Field experiments and phenotypic measurements

The field experiments with 185 DH lines of the YW population and two parental lines were conducted in randomized complete block designs, with three replicates except for 2010 in Wuhan where only two replicates were followed. The trials were conducted in three contrasting environments: (i) spring [two experiments, one each at Hezheng (Gansu province, China) and Xining (Qinghai Province, China)], (ii) semi-winter at Wuhan, representing the major rapeseed growing zone in China, and (iii) spring at Wagga Wagga (New South Wales, Australia; Supplementary Table 2, Supplementary Figure 1). The 81 diverse accessions were grown in Wuhan in 2013 and 2014 in randomized complete block designs with three replicates (Supplementary Table 2, Supplementary Figure 1). For Chinese environments, each plot was 3.0 m^2^ and 30 plants were planted in three rows with three replicates, with a distance of 40 cm between rows and 25 cm between individual plants. For Australian environment, trials were sown in pots under the birdcage at Wagga Wagga Agricultural Institute, NSW, Australia in 2013 and 2014. The trials consisted of two replications of four rows and 100 column pot array with each replication consisting of two rows and 100 column pot array. Five plants were sown per pot.

Seed yield, seven yield related traits (flowering time, pod width, pod length, seed number per pod, seed weight, pod number on main inflorescence, and length of main inflorescence) and six seed quality traits (protein content, oil content, erucic acid, linolenic acid, linoleic acid, and oleic acid) were investigated for QTL mapping in this study. The six seed quality traits (protein content, oil content, erucic acid, linolenic acid, linoleic acid, and oleic acid) were also measured on 81 diverse accessions of *B. carinata* that were used for GWAS. The seed yield and related traits were measured as described by Shi et al. (2009). Genetic variation in seed and oil quality traits was determined by Near-Infrared Reflectance Spectroscopy (NIR) method (Gan et al., 2003). Compared to other traits investigated in this study, only flowering time was determined in three different contrasting environments. We reanalyzed QTL for flowering time that were determined in a previous study (Zou et al., 2014), with the data collected from Australian Spring environment (WW13 and WW14).

### Statistical analyses

The broad sense heritability was calculated as *h*^*2*^ = σ_*g*_^*2*^/(σ_*g*_^*2*^ + σ_*ge*_^*2*^/*n* + σ_*e*_^*2*^/*nr*), where σ_*g*_^*2*^ is the genetic variance, σ_*ge*_^*2*^ is the variance representing genotype by environment interactions and σ_*e*_^*2*^ is the error variance, *n* is the number of environments and *r* is the number of replications. Analysis of variance (ANOVA) was performed using σ_*g*_^*2*^, σ_*ge*_^*2*^, and σ_*e*_^*2*^ estimated by using a Proc general linear model (GLM) in SAS software (SAS Institute Inc., 2000). Pearson correlations were calculated between phenotypic traits of interest (Weaver and Wuensch, 2013).

### QTL identification

A dense genetic linkage map of the YW DH population based on 4,031 presence-absence DArTseq markers corresponding to 1,366 unique loci (Zou et al., 2014) was utilized in this study. A total of 772 single nucleotide polymorphism (SNP) markers generated from the DArTseq were added to the genetic map by integrating in genetic bins (Supplementary Table 3). The present integrated map covered a genetic distance of 2,084 cM, with an average distance of 1.53 cM between loci and was subsequently used for QTL analysis.

QTL associated with various traits of interest were identified by the composite interval mapping model (Wang et al., 2012) using the software WinQTL cartographer version 2.5 (http://statgen.ncsu.edu/qtlcart/WQTLCart.htm). The criteria for identification of QTL were followed as described by Shi et al. (2009). The significant QTL (*P* = 0.05) with overlapping suggestive QTL (*P* = 0.5) named as “identified” QTL. The term “consensus” QTL was used for the same genomic interval identified for the same trait detected in different environments and was integrated by meta-analysis using the software BioMercator v4.2 (Goffinet and Gerber, 2000). We designated the “major” QTL which had *R*^2^ ≥ 20% and minor QTL which had *R*^2^ < 19.9%, as suggested by Collard et al. (2005). QTL that could be detected in more than one experiment were classified as “reproducible” QTL, while others were classified as “non-reproducible.”

### The alignment of identified QTL with previously published studies

We chose the reference genome of *B. napus* “Darmor-*bzh*” (Chalhoub et al., 2014), and the Tapidor/Ningyou (TN, *B. napus*) DH population as reference to compare QTL with those identified in YW DH population in this study. TN DH population has been extensively investigated previously and QTL associated with seed yield and its related traits and seed quality traits have been identified across different environments using genetic maps based on traditional makers (Shi et al., 2009) and a newly constructed SNP-based genetic bin map (Luo et al., under review). DArTseq markers underlying the identified QTL regions that were identified on the C genomes of YW DH population and TN DH population were aligned to the reference genome of *B. napus* “Darmor-*bzh*” (Chalhoub et al., 2014) by Blast using an *e*-value threshold of *e*^−10^ (Altschul et al., 1990) with ≥62 bp match length. The details of gene annotation for “Darmor-*bzh”* were cited from Körber et al. (2016). When the QTL regions of YW DH population could be aligned to the candidate gene for the same trait in the C genome of *B. napus*, we presumed there is a homologous candidate gene in the QTL region of *B. carinata*. When the QTL regions of YW DH population and TN DH population showed alignment to the same physical position on the reference genomes, we assumed the homologous QTL control genetic variation for trait of interest in YW and TN populations.

## Results

### Population structure, genetic relatedness, and LD analyses of *B. carinata*

**Figure 1:**
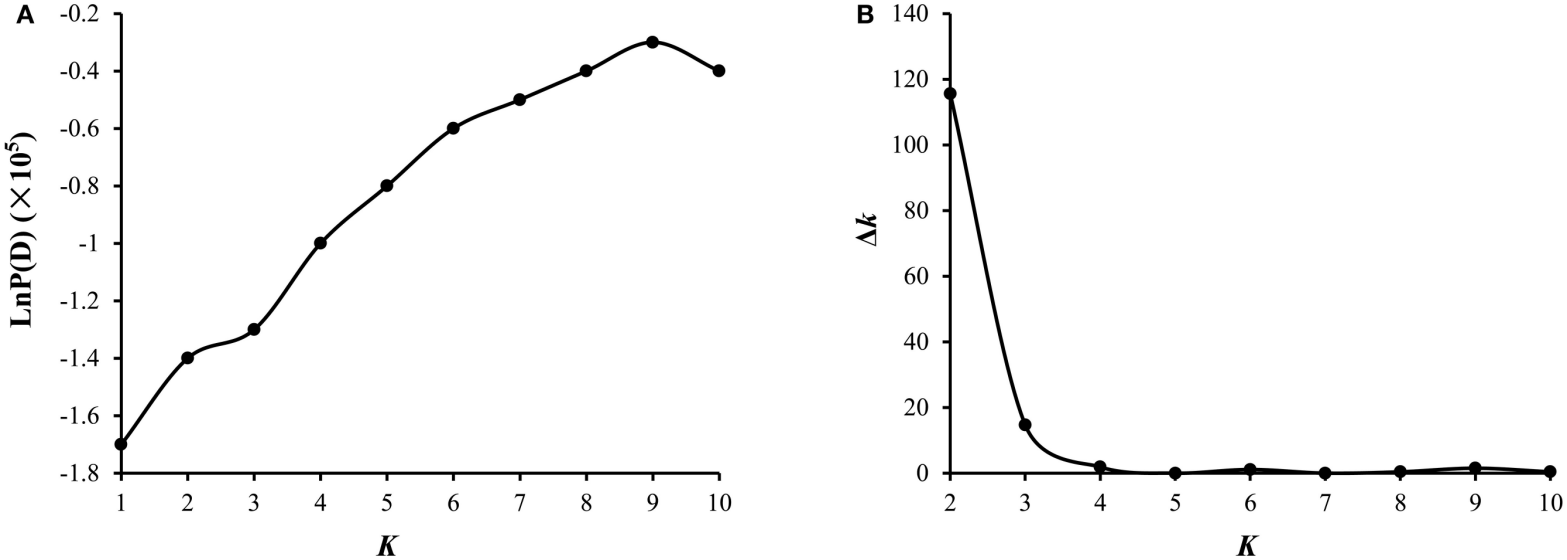
**Analysis of the population structure of 81 ***B. carinata*** accessions. (A)** Estimated LnP(D) of possible clusters (*K*) from 1 to 10. LnP(D), the log likelihood of the data in the STRUCTURE output. **(B)** Δ*K* based on the rate of change of LnP(D) between successive *K* as described by Evanno et al. (2005).

**Figure 2:**
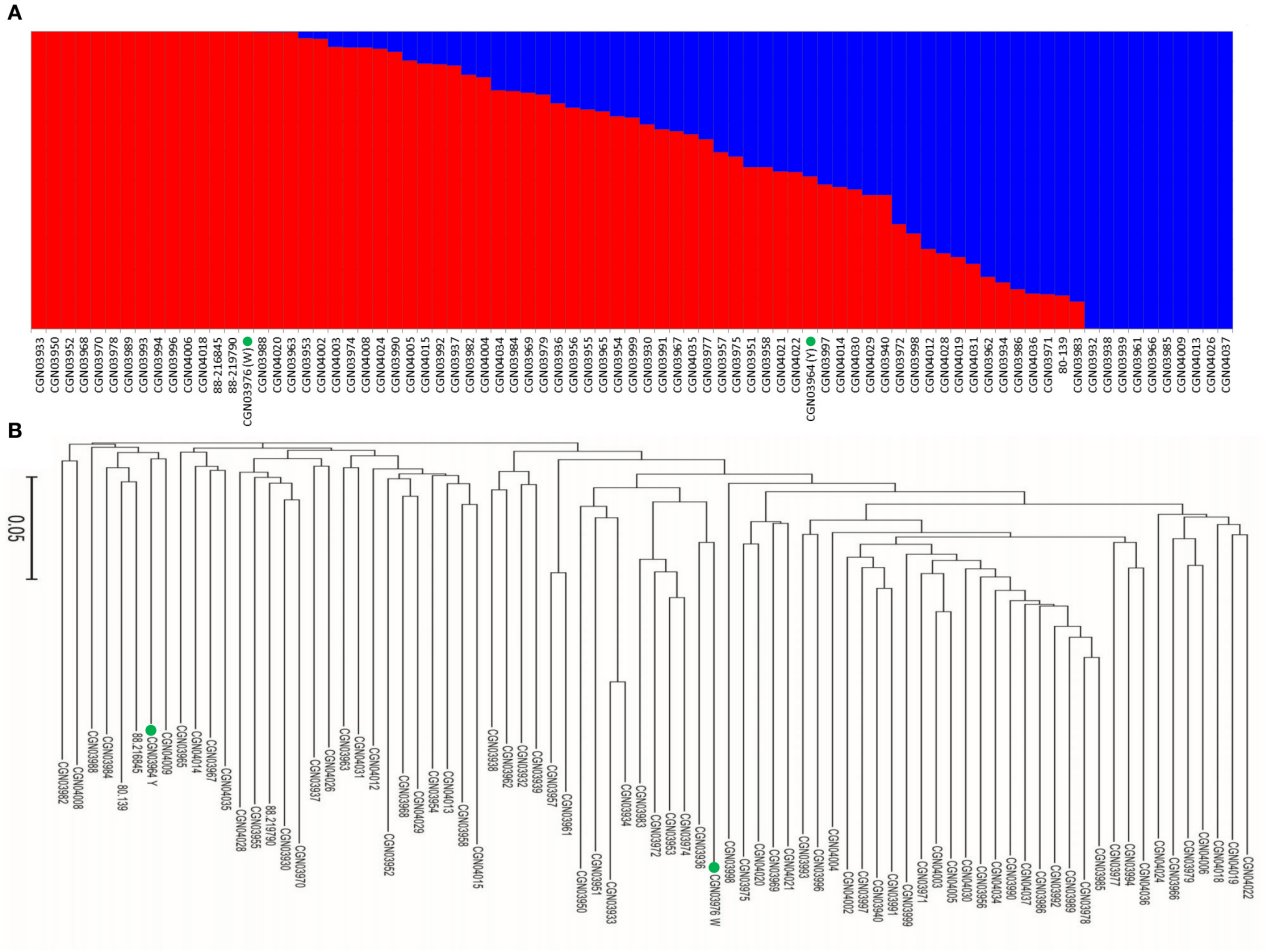
**Analysis of genetic diversity of 81 ***B. carinata*** accessions. (A)** The population structure of 81 accessions of *B. carinata*. *K* = 2. Two colors represent two sub-populations. **(B)** The neighbor-joining phylogenetic tree of 81 accessions of *B. carinata*. The two parents of YW DH population; Y-BcDH64 and W-BcDH76 were coded in green color.

A total of 54,510 polymorphic DArT-seq markers (38,396 presence-absence, also called *silico* DArTs and 16,114 SNPs) were identified in 81 accessions of *B. carinata*. After filtering, we selected 33,924 high quality markers that had overall call rates >90% and reproducibility >0.9, missing data of <10%, and MAF (minor allele frequency) >0.05 in the population, for the analyses of population structure and genetic diversity. The LnP(D) value decreased continuously with the change of *K* from 1 to 10, and the most significant change was observed when *K* increased from 1 to 2, Δ*K* also showed a peak at *K* = 2 (Figure 1, Supplementary Table 4). Accordingly, the 81 accessions could be divided into two major sub-populations (Figure 2A). Both parental lines of the YW DH population were found to represent different lineage as revealed by both population structure and NJ phylogenetic tree (Figure 2).

**Figure 3:**
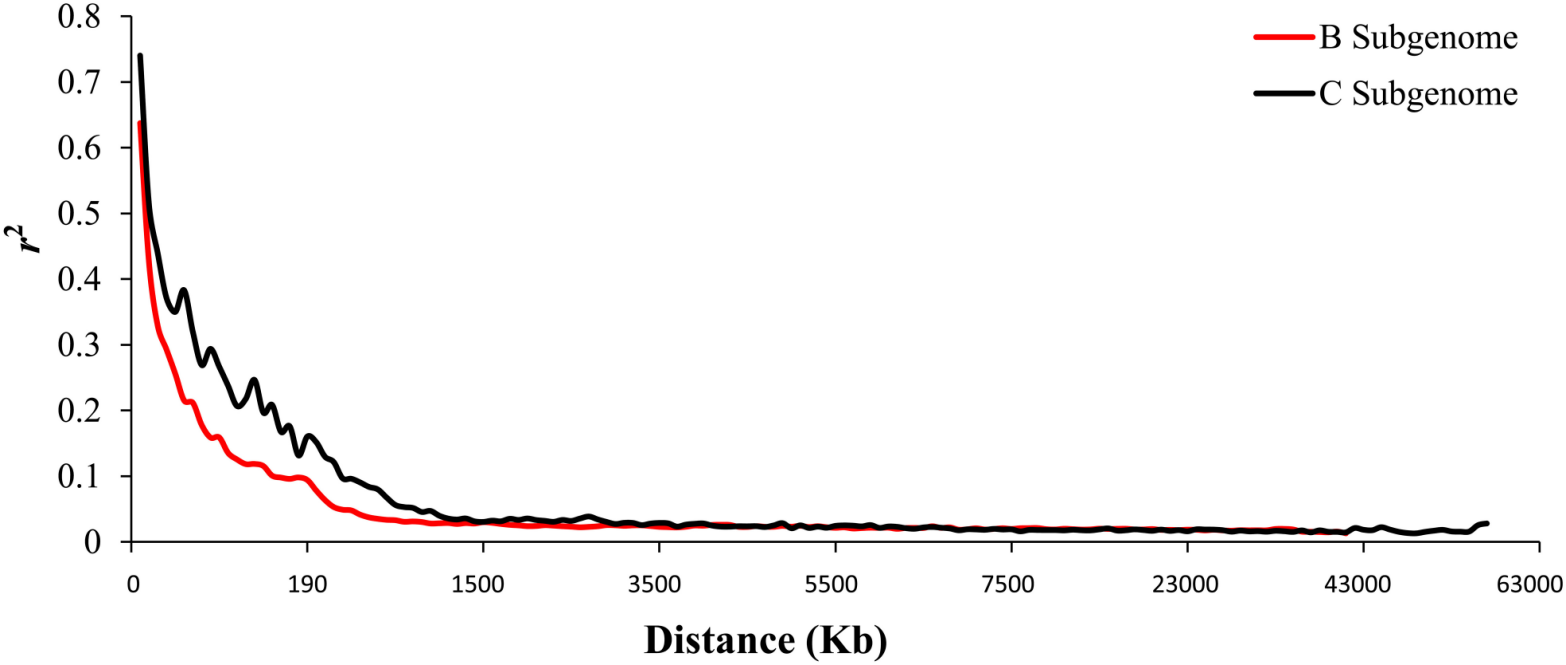
**The linkage disequilibrium (LD) pattern of the B and C subgenome estimated from 81 ***B. carinata*** accessions**. The red and black lines represent the distance of LD pattern in the B and C subgenome, respectively.

When we aligned the marker sequences (33,924) of the diverse *B. carinata* lines to the published C^n^ genome sequence of *B. napus* (Chalhoub et al., 2014) and the C° genome sequence of *B. oleracea* (Parkin et al., 2014), respectively, much more *B. carinata* markers sequence (1,458) could be uniquely aligned to *B. oleracea* (5,126) than that to *B. napus* (3,668) under the same parameters. Besides, the quality of the alignment to *B. oleracea* were much better, 87.87% of the alignments to the *B. oleracea* with over 65 bp match length, which is much higher than aligned to *B. napus* (0.65%). To avoid the potential subgenomic differentiation of the C genome between *B. carinata* and *B. napus*, we used the aligned results against its progenitor *B. oleracea*, as C genome reference. As a result, a total of 10,999 markers that uniquely aligned to the genomes of *B. nigra* (B genome) and *B. oleracea* (C genome) were then used to estimate the LD pattern of the diverse lines. Our result indicated that the C genome showed a longer LD decay (400 Kb) than the B genome (250 Kb) (Figure 3, Table 1). The LD decay distance varied across 17 chromosome, from 150 Kb (B4) to 650 Kb (C4, C6, and C9; Table 1). These results revealed significant differences in the level of LD between different chromosomes and subgenomes.

**Table 1:** **Summary of the polymorphic markers used for linkage disequilibrium estimation on the 17 chromosomes of ***B. carinata*****.

**Chromosome**	**Number of SNPs**	**Density of SNP (Kb/SNP)**	**LD decay^a^ (Kb)**
B1	659	46.9	250
B2	861	51.5	300
B3	949	46.8	250
B4	697	47.0	250
B5	799	48.9	350
B6	532	61.1	160
B7	532	71.6	160
B8	844	53.1	150
Average	734.1	53.4	250
C1	481	91.0	250
C2	449	117.8	190
C3	818	79.4	250
C4	648	82.9	650
C5	404	116.1	190
C6	615	64.8	650
C7	584	82.8	450
C8	547	76.3	170
C9	580	94.3	650
Average	569.6	89.5	400

a *LD decay means the physical distance on the genome when the average value of pairwise r^2^ decrease to 0.1*.

### Phenotype variation within the YW DH population across environments

Estimated means of parental lines, ranges and *h*^2^-values of traits measured in DH lines are given in **Table 3**. Both parental lines Y-BcDH64 and W-BcDH76 differed significantly for most traits measured across phenotypic environments (Table 2). W-BcDH76 had higher seed yield and 1,000 seed weight and early flowering time than Y-BcDH64. Estimated means for different seed quality traits in Y-BcDH64 were higher than W-BcDH76 in most environments except for oil content and oleic acid. Erucic acid was significantly different in the contrasting environments. Y-BcDH64 had higher erucic acid in semi-winter environment, but had a lower content in spring environment (Table 2).

**Table 2:** **Summary of the phenotypes of the YW DH population and its parents in different experiments**.

**Traits**	**Experiments investigated**	**Y-BcDH64 (Mean ± SD)**	**W-BcDH76 (Mean ± SD)**	**Range of YW DH population**
Seed yield (g/plant)	HZ12	2.90 ± 1.87	5.73 ± 1.63	1.22–19.43
	XN12	4.84 ± 0.87	12.28 ± 3.97	3.71–26.86
Flowering time (days)	WH09	187.30 ± 0.40	182.60 ± 0.40	167.00–194.00
	WH10	190.00	181.00	179.00–198.00
	HZ12	79.00 ± 1.00	77.33 ± 2.08	71.00–89.33
	XN12	89.33 ± 1.15	84.33 ± 0.58	81.00–97.33
	WW13	123.50 ± 1.91	118.00 ± 2.16	103.00–134.00
	WW14	140.25 ± 3.77	129.5 ± 1.00	109.00–151.00
Pod width (cm)	WH09	0.49 ± 0.04	0.74 ± 0.06	0.34–0.81
	HZ12	0.52 ± 0.03	0.75 ± 0.02	0.25–0.85
	XN12	0.39 ± 0.09	0.70 ± 0.04	0.33–0.73
Pod length (cm)	HZ12	7.80 ± 0.42	6.35 ± 1.20	4.93–8.31
	XN12	6.18 ± 0.54	5.33 ± 0.97	4.52–7.46
Seed number per pod	WH09	9.88 ± 2.17	13.66 ± 1.12	6.80–18.20
	HZ12	11.09 ± 1.88	11.75 ± 3.03	2.28–16.97
	XN12	11.70 ± 1.09	14.65 ± 1.38	5.68–17.25
	WH13	8.65 ± 0.31	10.75 ± 0.60	3.04–13.79
Seed weight (g)	WH09	2.80	3.48	2.09–4.53
	HZ12	3.25 ± 0.14	3.49 ± 0.08	2.58–4.77
	XN12	3.70 ± 0.42	4.16 ± 0.50	2.49–5.43
	WH13	3.42	4.30	1.44–5.04
Pod number of main inflorescence	WH09	16.70 ± 1.23	16.90 ± 1.04	8.10–24.30
Length of main inflorescence (cm)	WH09	27.12 ± 3.55	33.58 ± 3.38	16.40–41.76
Protein content (%)	WH09	33.89 ± 1.27	31.39 ± 0.67	27.34–35.85
	WH10	36.21 ± 1.27	35.18 ± 0.44	29.57–39.83
	HZ12	32.03 ± 1.86	28.83 ± 1.22	24.82–33.45
	XN12	26.34 ± 0.69	24.76 ± 0.14	20.51–29.40
	WH13	33.45 ± 0.41	31.66 ± 0.02	28.79–37.03
	WH14	30.86 ± 1.94	28.16 ± 1.36	23.85–32.57
Oil content (%)	WH09	32.19 ± 2.14	31.56 ± 1.16	24.34–41.17
	WH10	33.68 ± 1.91	32.28 ± 0.67	26.36–39.79
	HZ12	33.60 ± 2.40	33.81 ± 1.91	25.91–43.04
	XN12	36.00 ± 2.26	38.99 ± 1.75	32.94–48.44
	WH13	33.75 ± 0.21	35.58 ± 0.30	24.16–40.68
	WH14	36.88 ± 2.03	38.76 ± 1.36	30.03–46.35
Erucic acid (%)	WH09	21.41 ± 2.19	19.55 ± 1.08	4.84–30.57
	WH10	28.20 ± 0.85	23.68 ± 0.67	12.68–34.33
	HZ12	23.30 ± 2.09	24.30 ± 2.40	6.29–34.05
	XN12	24.99 ± 1.71	27.17 ± 1.87	14.84–36.72
	WH13	24.42 ± 0.90	24.79 ± 0.76	10.33–32.92
	WH14	33.40 ± 2.33	29.49 ± 1.75	19.27–41.33
Linolenic acid (%)	WH09	11.79 ± 0.88	10.97 ± 0.37	8.41–12.44
	WH10	12.50 ± 0.24	11.54 ± 0.53	10.08–13.75
	HZ12	13.08 ± 0.47	11.53 ± 0.70	9.84–13.29
	XN12	14.34 ± 0.23	12.68 ± 0.69	11.08–14.11
	WH13	12.43 ± 0.11	13.23 ± 0.18	10.79–14.25
	WH14	13.20 ± 0.60	12.13 ± 0.47	11.18–14.54
Linoleic acid (%)	WH09	22.54 ± 1.14	19.11 ± 0.88	18.16–24.74
	WH10	19.80 ± 0.43	16.22 ± 0.31	14.00–22.82
	HZ12	23.65 ± 1.34	18.73 ± 1.89	15.47–28.46
	XN12	22.07 ± 0.24	16.43 ± 0.77	14.91–23.40
	WH13	23.61 ± 0.15	20.34 ± 0.26	19.55–29.47
	WH14	25.16 ± 1.45	18.82 ± 1.02	18.52–24.98
Oleic acid (%)	WH09	26.53 ± 2.17	33.46 ± 1.54	17.92–42.92
	WH10	21.49 ± 1.77	32.87 ± 1.02	15.63–40.05
	HZ12	23.19 ± 2.70	28.86 ± 2.16	11.19–38.78
	XN12	16.76 ± 2.24	22.47 ± 1.05	6.47–33.74
	WH13	22.89 ± 0.92	17.05 ± 1.28	12.15–33.32
	WH14	15.78 ± 1.96	21.66 ± 1.56	2.33–28.65

**Figure 4:**
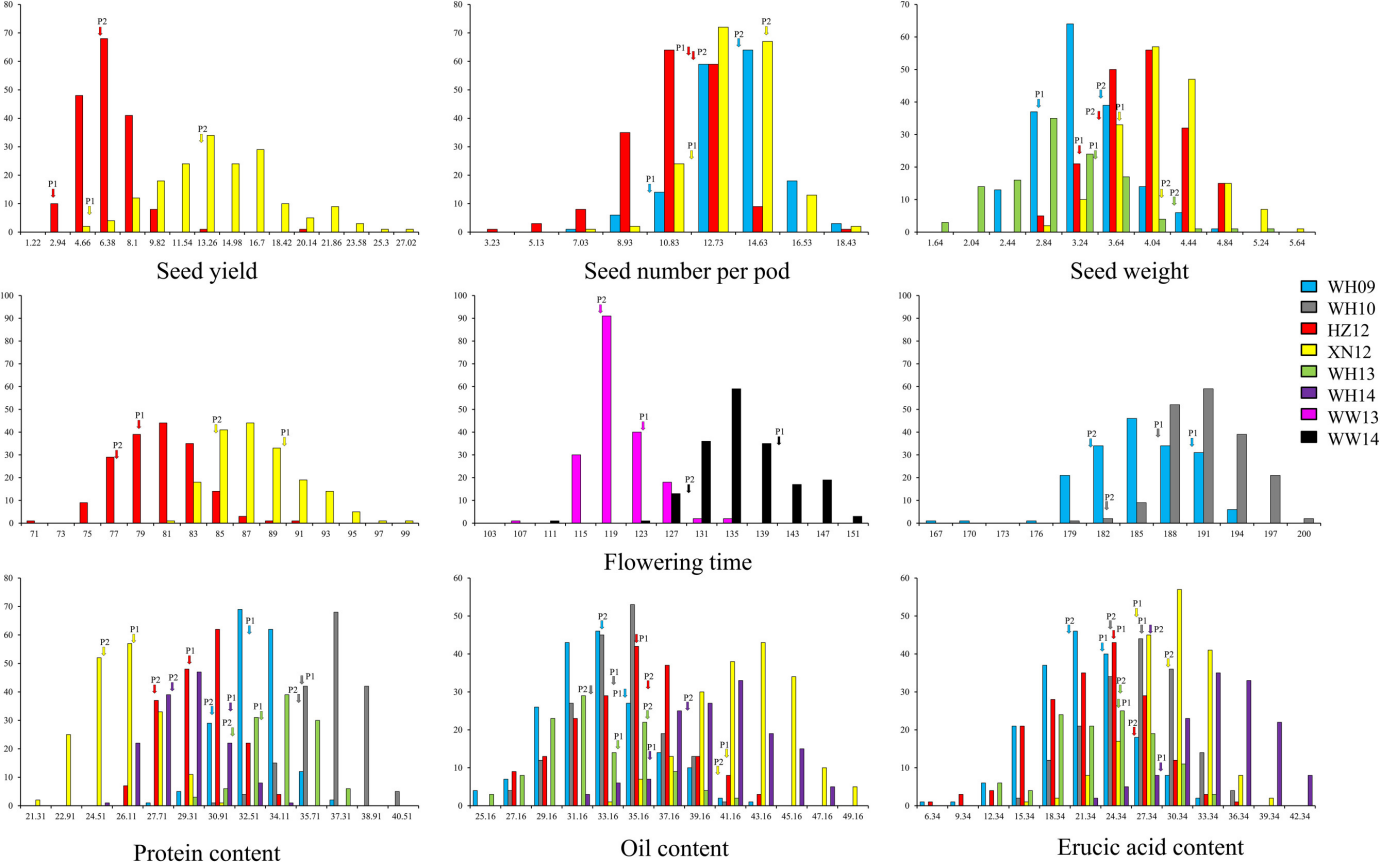
**The frequency distribution of representative traits in YW DH population**. Seed yield with three seed yield related traits and three seed quality traits were showed in this figure. P1 and P2 represent the two parents Y-BcDH64 and W-BcDH76, respectively. Transgressive segregation was observed in the population from all the experiments.

**Figure 5:**
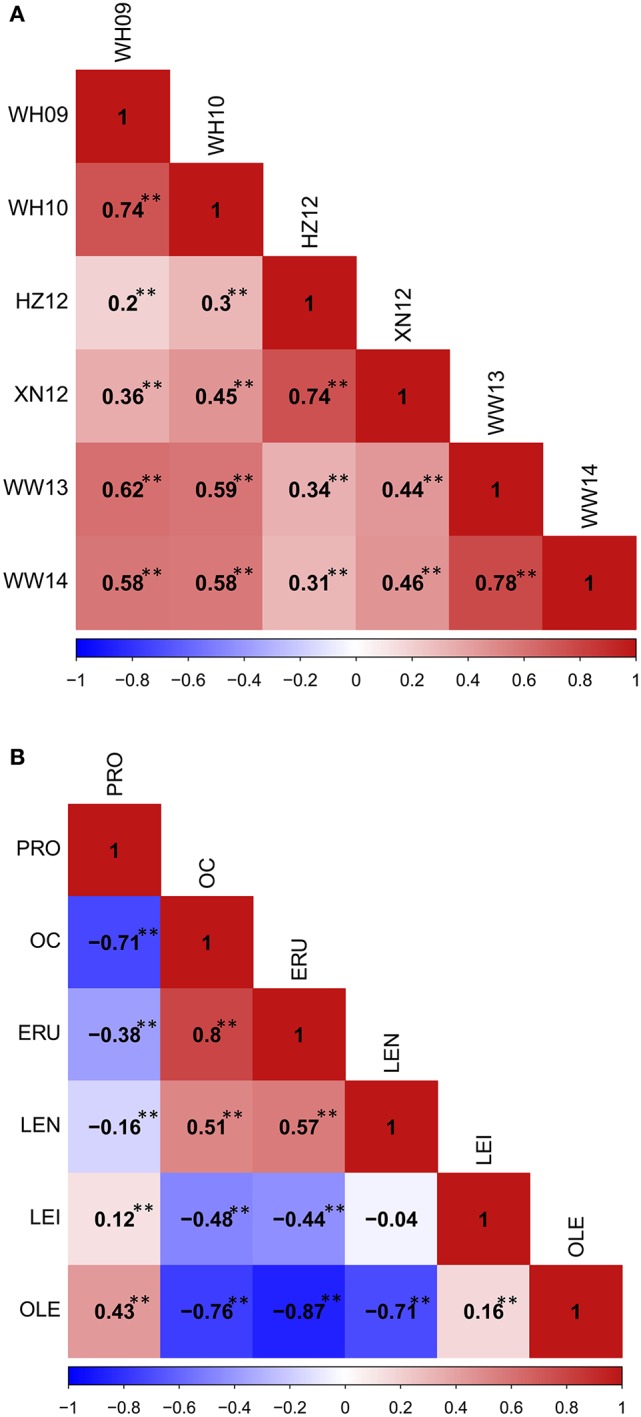
**The phenotypic correlation in YW DH population**. **(A)** The phenoytpic correlation of flowering time among different traits. **(B)** The phenotypic correlation among seed quality traits. ^**^P < 0.001.

A wide range of phenotypic variation was observed for all 14 traits between the parents and among the DH lines under different environments. Moderate to high *h*^2^ was observed in the YW population for 10 traits measured in more than one experiment, ranging from 15.59% for pod width (spring) to 90.79% for linolenic acid (spring) (Table 3). Frequency distributions for trait means clearly showed transgressive segregation for all the traits in the DH lines (Figure 4, Supplementary Figure 2). ANOVA on the investigated traits across different environments showed that “genotype (G),” “environment (E),” and “G × E” have significant effects for almost all of the traits except for the flowering time (at Wagga Wagga, Australia; Table 3). However, significant positive correlations for the flowering time were observed in different experiments, WH09 and WH10, HZ12, and XN12, WW13, and WW14 (Figure 5A). The correlation coefficient for the flowering time observed between semi-winter (China) and spring (Australia) experiments was higher than that between semi-winter (China) and spring (China), and spring (China) and spring (Australia).

**Table 3:** **AVOVA for the investigated agronomic traits and seed quality traits in YW DH population across environments**.

**Phenotype**	**Contrasting environment**	**Genotype^a^**	**Environment^a^**	**Genotype × Environment^a^**	**Error**	***h*^2^ (%)**
Seed yield	Spring (China)	3095.64 (181)^**^	10790.85 (1)^**^	3088.98 (170)^**^	2328.73 (443)	15.59
Flowering time	Semi-Winter (China)	10105.58 (172)^**^	–	–	2042.50 (344)	–
	Spring (China)	9065.44 (180)^**^	13377.43 (1)^**^	1062.74 (179)^**^	3340.17 (670)	88.21
	Spring (Australia)	15999.25 (181)^**^	49170.73 (1)^**^	3116.02 (181)	6027.50 (364)	80.52
Pod width	Semi-Winter (China)	2.71 (178)^**^	–	–	0.10 (328)	–
	Spring (China)	3.21 (180)^**^	0.03 (1)	1.85 (179)^**^	3.77 (638)^**^	41.90
Pod length	Spring (China)	267.99 (180)^**^	151.48 (1)^**^	95.15 (178)^**^	264.90 (648)	64.1
Seed number per pod	Semi-Winter (China)	2072.25 (178)^**^	1468.03 (1)^**^	662.80 (117)^**^	643.98 (474)	51.34
	Spring (China)	1762.17 (180)^**^	1453.40 (1)^**^	1358.70 (179)^**^	3021.35 (619)	22.47
Seed weight	Semi-Winter (China)	116.14 (173)^**^	–	–	33.30 (344)	–
	Spring (China)	173.51 (179)^**^	12.87 (1)^**^	30.47 (170)^**^	54.43 (542)^**^	81.51
Pod number of main inflorescence	Semi-Winter (China)	9390.10 (181)^**^	–	–	334.07 (652)	–
Length of main inflorescence	Semi-Winter (China)	23412.85 (181)^**^	–	–	2840.65 (665)	–
Protein content	Semi-Winter (China)	3126.25 (184)^**^	13000.33 (3)^**^	1112.46 (426)^**^	429.10 (960)	84.63
	Spring (China)	2028.03 (180)^**^	4248.64 (1)^**^	1095.10 (179)^**^	2710.48 (650)	45.70
Oil content	Semi-Winter (China)	12088.16 (184)^**^	15350.10 (3)^**^	4727.00 (423)^**^	1719.61 (960)	82.99
	Spring (China)	8867.98 (180)^**^	13689.62 (1)^**^	2795.11 (177)^**^	6647.79 (645)	67.95
Erucic acid	Semi-Winter (China)	25736.21 (183)^**^	39613.30 (3)^**^	7024.24 (412)^**^	3.58 (943)	87.88
	Spring (China)	14735.67 (180)^**^	11994.34 (1)^**^	5438.31 (179)^**^	9144.75 (637)	62.89
Linolenic acid	Semi-Winter (China)	570.89 (184)^**^	1026.32 (3)^**^	230.47 (423)^**^	89.50 (955)	82.44
	Spring (China)	452.00 (180)^**^	122.10 (1)^**^	42.40 (179)^**^	103.98 (640)	90.79
Linoleic acid	Semi-Winter (China)	2854.02 (184)^**^	6285.57 (3)^**^	1209.16 (426)^**^	595.78 (957)	81.70
	Spring (China)	2903.28 (181)^**^	1289.29 (1)^**^	1353.14 (178)^**^	453.57 (604)	52.87
Oleic acid	Semi-Winter (China)	32705.67 (183)^**^	54210.20 (3)^**^	10592.41 (416)^**^	3967.23 (930)	85.75
	Spring (China)	25640.61 (180)^**^	12587.76 (1)^**^	6087.14 (179)^**^	9207.50 (628)	76.13

a *Sum of square from two-way ANOVA, the number in bracket indicates the df (degrees of freedom)*.

** *P < 0.0001*.

– *Not determined*.

Phenotypic correlations among seed quality traits were also calculated (Figure 5B). The correlation between oil content and oleic acid, and oil content and linoleic acid showed the significant negative correlation, but a significant positive correlation was observed between oil content and erucic acid, as well as oil content and linolenic acid. Oleic acid showed positive correlation with linoleic acid, but significant negative correlation with linolenic acid and erucic acid. These results showed that the different fatty acid components in the seeds correlate with high erucic acid.

### Identification of QTL for seed yield and its related traits

**Figure 6:**
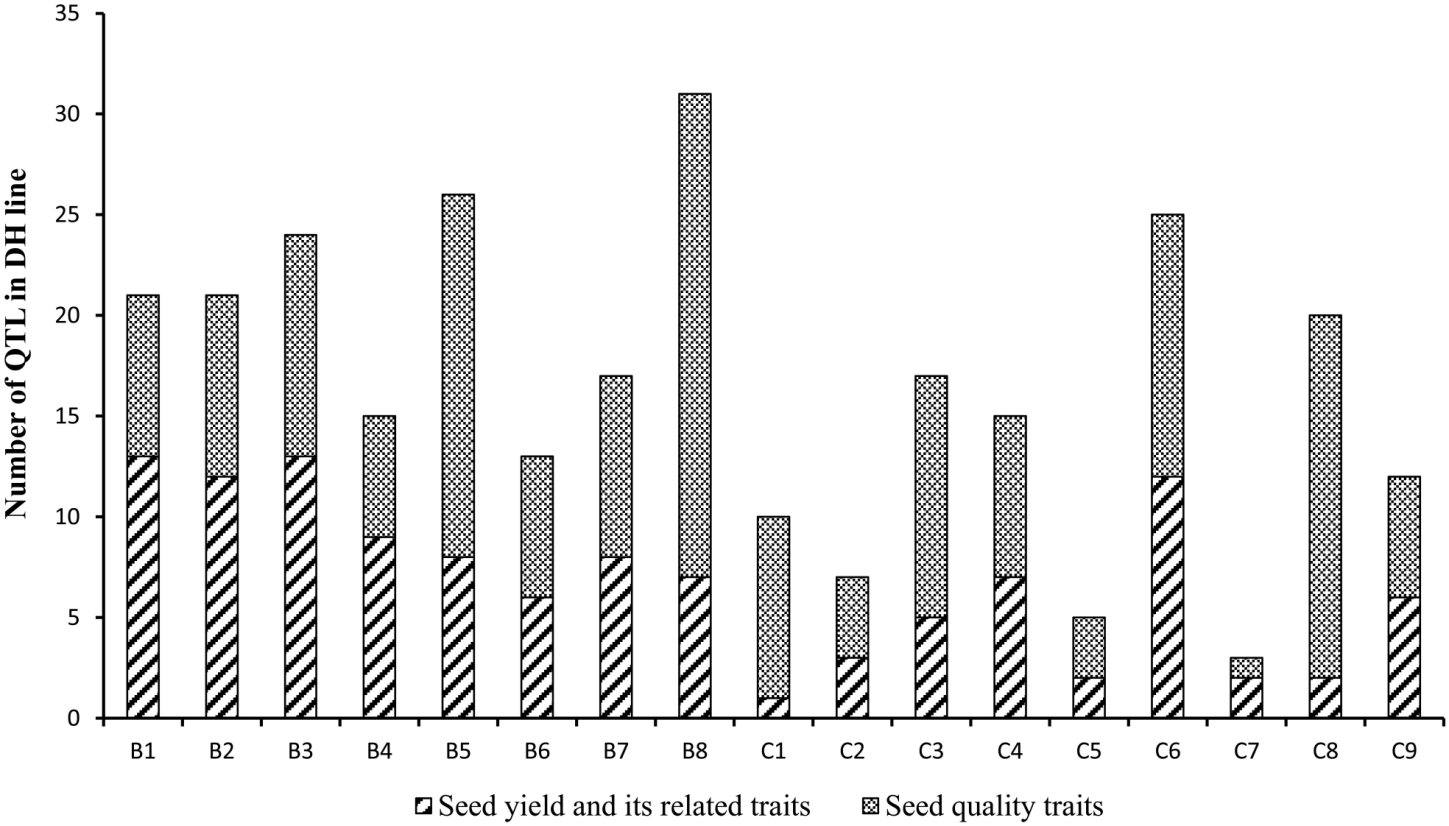
**The number of the consensus QTL across the 17 linkage groups of YW DH population**. The boxes with diagonal lines and cross lines represent the QTL number for seed yield with its related traits and seed quality traits, respectively.

A total of 150 identified QTL for seed yield, flowering time, pod width, pod length, seed number per pod, seed weight, pod number of main inflorescence, and length of main inflorescence were identified in the YW DH population (Supplementary Table 5). After binning QTL which were repeatedly detected from different experiments for the same traits, a total of 116 consensus QTL were identified for the eight agronomic traits. Of these, 76 consensus QTL were detected in B genome while 40 were detected in C genome. The most number of consensus QTL were identified in chromosomes B1 and B8 (13 each), while the least number of consensus QTL (1) were identified in C1 (Figure 6, Supplementary Figure 3).

Six consensus QTL for seed yield were identified (only measured in two experiments, HZ12 and XN12, because of the high pod shattering) on chromosomes B2, B4, B6, C4, and C9, explaining phenotypic variation ranging from 4.2 to 16.7%. The parent W-BcDH76 contributed favorable alleles of the QTL on B2, B4, C4, and C9, whereas parent Y-BcDH64 contributed favorable alleles of two consensus QTL for seed yield (*qSY.B6-1, qSY.B6-2*) located on B6 chromosome (Supplementary Table 5). None of the consensus QTL was consistently detected across experiments. But four of the six consensus QTL overlapped with the QTL accounting for other yield related traits. For instance, *qSY.B4-1* and *qSY.C9-1* for seed yield were overlapped with the QTL for seed number per pod (*qSN.B4-1, qSN.C9-1*), and *qSY.B6-1* was overlapped with the QTL for seed weight (*qSW.B6-1*) (Supplementary Figure 3).

**Figure 7:**
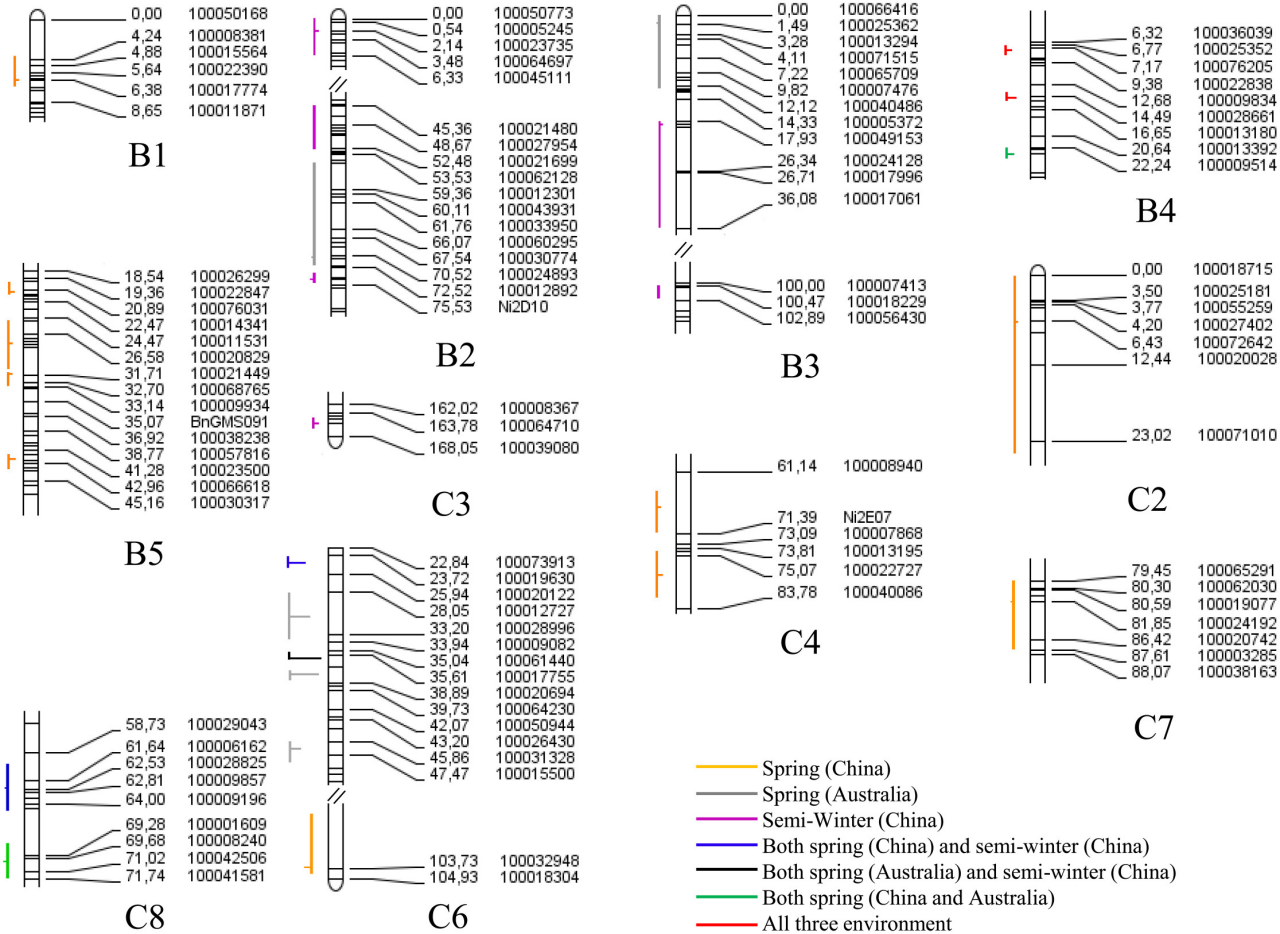
**The QTL controlling the flowering time in multiple environments of the YW DH population**. All of the QTL are arranged to the left of each linkage group with vertical lines representing confidence interval, and the peak position of QTL is shown with a horizontal lines. The horizontal lines located to the left or right of the vertical lines represent the positive or negative additive effect. The lines with different color represent different environments.

For flowering time, a total of 28 consensus QTL were identified on 11 chromosomes in three macro-environments (Australian spring, Chinese spring, and Chinese semi-winter; Figure 7). The identified QTL (*qFT35*, C6) integrated in the consensus QTL could explain up to 59.40% of the phenotypic variation in flowering time (Supplementary Table 5). A total of three consensus QTL, all of which located in C6, were identified as “major QTL” individually explaining phenotypic variation over 20% (Table 4). For instance, the consensus QTL, *qFT.C6-3* on chromosome C6 within 1 cM interval (genetic position from 35.5 to 36.5 cM) explained the maximum phenotypic variation (from 28.0 to 59.4%). Of the three major QTL, *qFT.C6-1* was identified in the semi-winter environment of China with high *R*^2^ (27.13% in experiment WH09 and 42.36% in experiment WH10), but low *R*^2^ (2.35% in experiment XN12) identified in the spring environment of China. Among the 28 total QTL for flowering time, seven of them (*qFT.B4-1, qFT.B4-2, qFT.B4-3, qFT.C6-1, qFT.C6-3, qFT.C8-1*, and *qFT.C8-2*) were identified in at least two macro-environments (Supplementary Table 5). Two of the seven QTL (*qFT.B4-1* and *qFT.B4-2*) were identified in all the three environments, and both were located in B4 chromosome. Four of the seven consensus QTL, *qFT.B4-3, qFT.C8-2*, and *qFT.C6-1, qFT.C8-1* were identified in the two spring macro-environments (Australian spring and Chinese spring) and two Chinese macro-environments (Chinese semi-winter and Chinese spring), respectively. While, the last one of the seven consensus QTL, *qFT.C6-3* was identified in both of the Chinese semi-winter environment and Australian spring environment. These results indicated the differential genetic effects of the QTL in response to environment.

**Table 4:** **Major QTL accounting for the seed and oil quality traits in the YW DH population**.

**Consensus QTL**	**Trait**	**Linkage group**	**Lod score**	***R*^2^ (%)**	**Peak position**	**Start**	**End**	**Macro-environment**	**Experiments code**
*qPNM.B3-1*	Pod number of main inflorescence	B3	17.90	28.41	25.01	20.60	30.80	Semi-Winter	WH09
*qERU.C4-1*	Erucic acid	C4	3.42–21.80	5.69–32.60	132.02	130.35	133.7	Both	WH09|XN12|WH10|WH14|HZ12
*qOLE.C4-1*	Oleic acid	C4	15.26–21.56	27.28–35.85	127.72	126.23	129.21	Both	XN12|WH09
*qOLE.C4–2*	Oleic acid	C4	4.22–35.44	10.21–50.43	136.39	134.82	137.96	Both	WH13|WH10|XN12|HZ12|WH14|WH09
*qLEN.C4-1*	Linolenic acid	C4	4.08–31.15	7.47–46.19	137.02	135.72	138.33	Both	WH13|WH10|XN12|WH14|HZ12|WH09
*qFT.C6-1*	Flowering time	C6	15.70–23.33	27.13–42.36	24.62	23.84	25.39	Both	XN12|WH10|WH09
*qFT.C6-3*	Flowering time	C6	17.57–41.06	28.04–59.46	35.98	35.46	36.50	Both	WH10|WH09|WW14
*qFT.C6-4*	Flowering time	C6	21.70	33.10	38.01	37.60	38.90	Spring	WW13
*qSW.C6-1*	Seed weight	C6	11.89	20.18	39.71	39.30	41.70	Semi–Winter	WH09

### Identification of QTL for seed quality traits

A total of 296 identified QTL were detected for protein content, oil content, erucic acid, linolenic acid, linoleic acid and oleic acid in the seeds of the YW DH population (Supplementary Table 5). These identified QTL could be integrated as 166 consensus QTL and localized across the 17 chromosomes, explaining phenotypic variation varying from 0.2 to 50.4%. Four of these QTL, located in C4, explained ≥20% of the phenotypic variation and therefore were designated as major QTL (Table 4). Consistent with the distribution of QTL for seed yield and yield related traits in subgenomes, more QTL were identified in the B genome (92) compared with that in the C genome (74) for seed quality traits (Figure 6). In chromosome B8, the most number of consensus QTL (24) were detected for seed quality traits, whereas the least (1) were identified in chromosome C7.

Among the six seed quality traits, the maximum number of consensus QTL were identified for oil content (34) and the minimum were identified for protein content (20). The QTL for oil content were detected on all chromosomes of *B. carinata* genome except B9 and C7 (Supplementary Table 5). The *R*^2^ explained by these QTL ranged from 2.67% (*qOC.C5-1* on C5) to 10.4–23.4% (*qOC.C4-1* on C4). Nine consensus QTL for oil content were repeatedly detected in at least two experiments. The alleles from the parent W-BcDH76, at the QTL located in chromosome B5, C3, C4, C5, C6, C8, and C9, contributed to the increase of oil content in YWDH population.

A total of 30 consensus QTL for erucic acid were detected on all chromosomes of *B. carinata* except C2 and C7 (Supplementary Table 5). The QTL *qERU.C4-1* on C4, accounted for the maximum phenotypic variation (5.7–32.6%) for erucic acid. The alleles from the parent Y-BcDH64 for the four QTL (*qERU.B5-1, qERU.C3-2, qERU.C9-1*, and *qERU.C9-2*) contributed negatively to increase the erucic acid.

Using the phenotypic data of six seed quality traits investigated for the 81 diverse lines in two experiments, we also identified the markers accounting for genetic variation in seed quality traits by GWAS and compared with the results revealed by linkage analyses. A total of 12,030 DArT-seq markers with known physical position were used for GWAS. According to the QQ plots, the suitable model for each trait was selected. Seven markers were found to be significantly associated with five traits (Table 5). Linoleic acid showed a very limited phenotypic variation among diverse lines. As a result, we could not detect any association for this trait. One of the seven markers (5121285) was significantly associated for three traits (PRO-WH13, OC-WH13, and ERU-WH13). Two markers 5868483-1S and 5121285 showed significant association with linolenic acid (LEN-WH14) and erucic acid (ERU-WH13), respectively. Both these markers also located in the QTL region for the same traits in the YW DH population (Table 5). The marker 5,121,285 was also aligned to the homologous QTL region for oil content of the TN DH population of *B. napus* (Luo et al., under review).

**Table 5:** **The DArTseq markers significantly associated with the seed quality traits detected using GWAS in diverse ***B. carinata*** accessions**.

**Trait**	**Marker name**	**Marker sequence**	**Chromosome**	Physical position aligned to reference sequence (Mb)^**^	***P*-value**	***R*^2^ (%)**	**Comparison with the QTL detected for the same trait of the YW DH and TN DH population**
PRO-WH13^*^	5121285	TGCAGATCTCTGACAAACAAACTGAAACGTCTCG AAAAATTCCATTGATTGAGGGAATCCTTCCACTGA	C8	68.68	1.46E-06	29.27	–
OLE-WH13	3112643S	TGCAGTTGCGAAATTCGGAAAGTATAAAGCCCTC GGTTTCCAACTCAGTACCTGCACCATTGTTACAGA	B7	101.00	1.42E-05	27.18	–
OLE-WH13	3155095	TGCAGCGGAGAAATCGTTGAAGATCCGGCTTCAG TTGAATCATACTTCTAACAGTGGAAGAGAATCTCT	C4	134.73	7.26E-05	19.92	–
OLE-WH14	5859309S	TGCAGAAAGCACCTCATGAGGTTACTCCTAAGAA TCGGTTGAGTCAAGTGTTGGGGGTGTTCGACAAGT	B2	99.84	3.21E-05	31.83	–
OLE-WH14	5855685S	TGCAGTTTTCTTACTCTTTGGCCTTCTTTTGCTTC AGCTCAAGCCACATCCTGATCAGAAATCCATTAC	B3	94.01	3.91E-05	31.25	–
OLE-WH14	5851108S	TGCAGATACTCCAGCTATCAACCCAGTACTCGGTT TCGAACTCTTGTTACGCGCATGAAAAACAGCATC	B3	103.16	4.77E-05	30.98	–
OC-WH13	5121285	TGCAGATCTCTGACAAACAAACTGAAACGTCTCG AAAAATTCCATTGATTGAGGGAATCCTTCCACTGA	C8	68.68	2.72E-06	27.40	TN DH; Luo et al., under review
LEN-WH14	5863483-1S	TGCAGTAAGAAACATGGCACATAGTGGTCAGCTG AATCACAGCCATTGCAGCATCTCCTGATGACTGGT	B3	12.59	5.67E-05	22.08	YW DH; *qLEN8*
ERU-WH13	5121285	TGCAGATCTCTGACAAACAAACTGAAACGTCTCG AAAAATTCCATTGATTGAGGGAATCCTTCCACTGA	C8	68.68	8.13E-05	20.38	YW DH; *qERU51*

* *The trait name is shown as trait abbreviations plus the experiment code. PRO, OLE, OC, LEN and ERU means protein content, oleic acid, oil content, linolenic acid and oleic acid, respectively. WH13 and WH14 means the experiment conducted in Wuhan, China in 2013 and 2014, respectively*.

** *The reference sequence of B genome and C genome are referred to the published sequence of B. nigra (Yang et al., 2016) and B. napus (Darmor-bzh), respectively*.

### Alignment of QTL regions accounting for different traits and the comparison with homologous QTL region in the C subgenome of *B. napus*

A majority (78%) of the consensus QTL accounting for different traits in the YW DH population investigated could be overlapped with each other (Supplementary Figure 3, Supplementary Table 5). For example, one of the QTL accounting for seed weight (*qSW.B4-1*) was overlapped with the one for flowering time (*qFT.B4-1*) in B4 chromosome. Whereas, the other QTL for seed weight (*qSW.B5-2*) was overlapped with the one for pod width (*qPW.B5-1*) in B5 chromosome. In C4 chromosome, there were five overlapping QTL, *qPRO.C4-1, qLEI.C4-2, qOLE.C4-2, qLEN.C4-1*, and *qOC.C4-1* for protein content, linoleic acid, oleic acid, linolenic acid, and oil content, respectively (Supplementary Figure 3, Supplementary Table 5).

**Figure 8:**
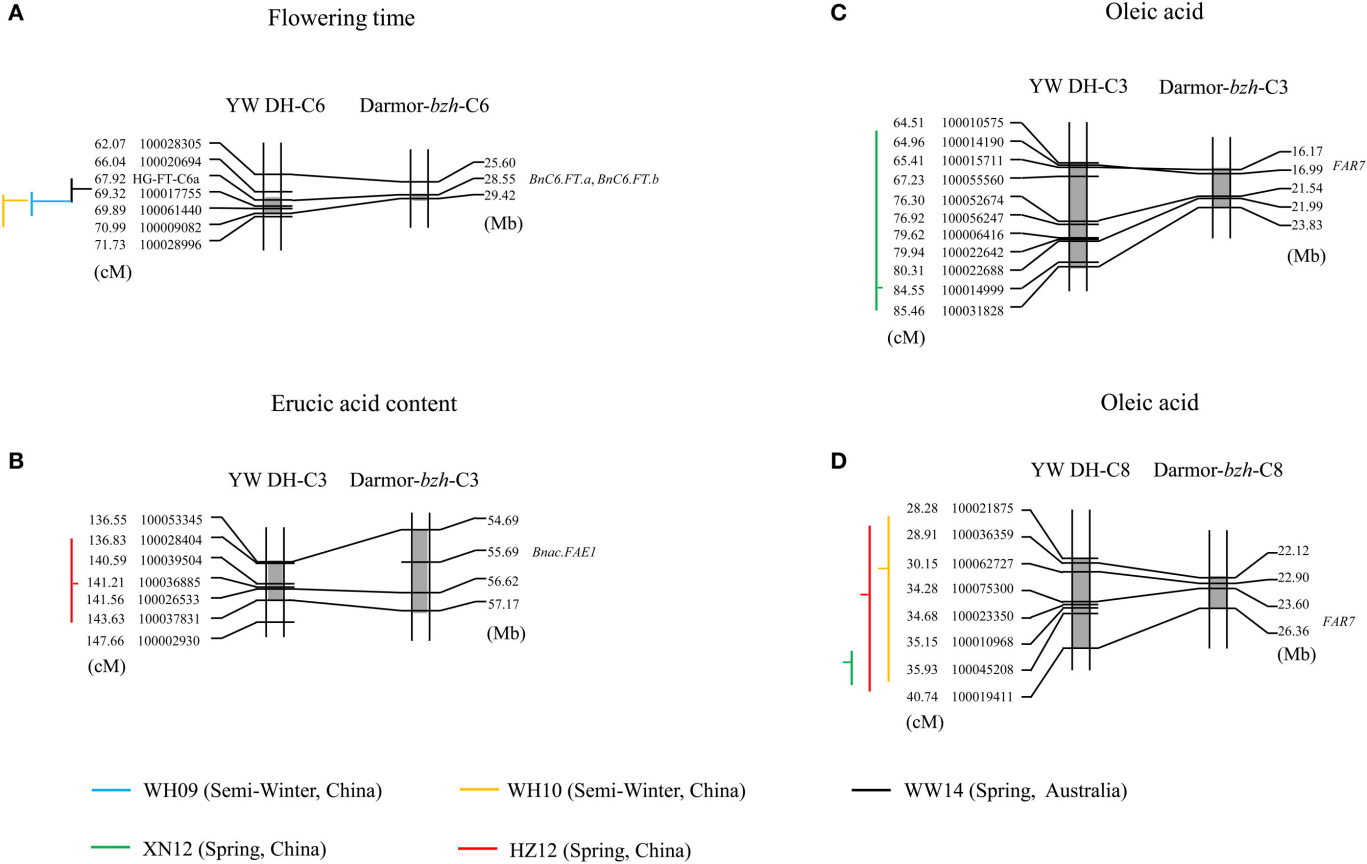
**The QTL regions identified in C genome of YW DH population and the physical position of genes in Darmor-***bzh*** (***B. napus***)**. **(A,B)** represent the alignment of the QTL for flowering time in chromosome C6 and erucic acid in chromosome C3, respectively. **(C,D)** represent the alignment of the QTL for oleic acid in chromosome C3 and C8, respectively. The lines with blue, yellow, black, green, and red represent the QTL in the experiments of WH09, WH10, WW14, XN12, and HZ12, respectively. The gray shadow box showed the QTL region in the chromosomes. The vertical lines and horizontal lines represent the confidence interval and the peak position of QTL, respectively. The horizontal lines located to the right of the vertical lines means the negative additive effect. The parent W-BcDH76 contributes the positive effect in the YW DH population.

To determine whether the homologous QTL genomic regions of *B. carinata* controlling variation for yield related traits and seed quality traits present in the C genome could also modulate genetic variation for these traits in the other oilseed *Brassica* crop containing C genome, we compared and aligned the QTL regions of YW DH population to the physical position of those QTL regions of TN DH population and candidate genes predicted in *B. napus* (the reference genome Darmor-*bzh*). The QTL accounting for flowering time, erucic acid and oleic acid of *B. carinata* could be aligned to the candidate genes of the *B. napus* for the three traits (Figure 8). For example, the genetic intervals of three identified QTL for flowering time (*qFT34, qFT35* and *qFT36*) were aligned to the physical positions 28.55–29.42 Mb on chromosome C6 of the reference genome of Darmor-*bzh* (Figure 8A). There were two genes controlling flowering time (*BnC6.FT.a* and *BnC6.FT.b*; Wang et al., 2009) located in the 28.55 Mb regions on C6 chromosome of Darmor-*bzh*. This suggested that both homoeologues of *FLOWERING LOCUS T* gene are likely candidates for flowering time variation in YW DH population of *B. carinata*. The QTL accounting for erucic acid (*qERU37*) in C3 chromosome of YW DH population was aligned to the gene *BnaC.FAE1* of *B. napus* ((Körber et al., 2016); Figure 8B). The QTL accounting for oleic acid in C3 (*qOLE25*) and C8 (*qOLE47, qOLE48*, and *qOLE49*) of the YW DH population were aligned to the gene *FAR7* gene in C3 and C8 of oleic acid in *B. napus* (Körber et al., 2016), respectively.

Additionally, a total of 17 QTL detected in the C genome of the YW DH population could also be aligned to the homologous QTL region for the same traits in the C genome of *B. napus* (Supplementary Table 5). For example, the identified QTL (*qFT34, qFT35*, and *qFT36*) for flowering time was aligned to the homologous QTL region in the TN DH population (Luo et al., under review). Three QTL (*qSW26, qSW27*, and *qSW31*) for seed weight and one QTL (*qPRO28*) for protein content were also aligned to the homologous QTL region of TN DH population. For the erucic acid, ten of the identified QTL (*qERU37, qERU49* to *qERU56, qERU59*) detected in C3, C6, and C8 chromosomes could be aligned to the homologous QTL region in the C genome of *B. napus*.

## Discussion

Development of high yielding varieties of *B. carinata* with canola quality oil/biodiesel properties is one of the important goals of some *Brassica* improvement programs. To utilize genetic diversity present in *B. carinata* accessions originated from Ethiopia and improve this species itself, it was important to evaluate the extent of genetic diversity and to identify the QTL associated with the important agronomic and seed quality traits.

Assessment of genetic diversity in any species is crucial for genetic improvement programs. This has been accomplished using morphological, biochemical (isozymes) and molecular markers in various crops. In *B. carinata*, different marker systems such as AFLP, and SSR markers have been utilized to analyse genetic diversity (Warwick et al., 2006; Jiang et al., 2007). In this study, we utilized high quality 33,924 DArTseq markers to analyse the genetic diversity. Our results on population structure revealed that 81 accessions could be grouped into two sub-populations (Figure 1). These results suggested that a limited genetic diversity exist in *B. carinata* accessions originated from different regions of Ethiopia- the center of genetic diversity of this species.

We found that the LD decay in *B. carinata* varied across chromosomes, consistent with previous findings in *B. napus* (Qian et al., 2014; Liu et al., 2016) and *B. oleracea* (Pelc et al., 2015). The LD in the C subgenome (0.17–0.65 Mb) decayed longer than that of the B subgenome (0.15–0.35 Mb) of *B. carinata*. A previous study in *B. napus* has shown that the LD decay of the C subgenome was also longer than that of the A subgenome (Liu et al., 2014). Long LD decay in the C subgenome of *B. napus* and *B. carinata* may be due to the high level of gene conservation which could have resulted from limited recombination or due to large segmental structural variation. However, the distance of LD decay (1.12–8.50 Mb) in the C subgenome of *B. napus* presented previously (Qian et al., 2014; Liu et al., 2016) seems to be much longer than that of the C subgenome of *B. carinata* (0.17–0.65 Mb) investigated in this study, and the size of the block with strong linkage disequilibrium in C genome also seems much longer in *B. napus*. But the distance of LD decay (0.6 Mb) in C subgenome of *B. oleracea* (Pelc et al., 2015) seems to be similar to *B. carinata*. Additionally, when we aligned the marker sequences (33,924) of the *B. carinata* diverse lines to the published C genome sequence of *B. napus* (Chalhoub et al., 2014) and the C genome sequence of *B. oleracea* (Parkin et al., 2014), significantly more *B. carinata* markers sequence could be uniquely aligned to *B. oleracea* (5,126) than that to *B. napus* (3,668) under the same parameters. These results may indicate that the C subgenome of *B. carinata* has substantially differentiated from the C subgenome of *B. napus*, and thus has great potential to broaden the genetic diversity of the C subgenome of *B. napus*.

A wide range of phenotypic variation was observed for all 14 traits among the DH lines under different environments and showed significant environment interactions (Figure 4, Supplementary Figure 2). The flowering time was shorter in spring environment than that in semi-winter environment as expected. The seed weight of lines grown in spring environment was heavier than that presented in semi-winter environment. The seed quality traits were also significantly affected by environments. For example, a higher oil content and erucic acid were observed in spring environment than that in semi-winter environment. This may have resulted from the spring-type habit of *B. carinata* which is more adapted under spring type environment. Due to the late maturity in semi-winter environment, the high temperature may adversely affect seed yield and quality attributes. Therefore, shortening the flowering time of *B. carinata* to adapt to the semi-winter environment would increase the seed yield and improve the seed quality of this species. In the YW DH population (high erucic acid), the correlations among the oil content and its related fatty acid content was different with that observed in the TN DH population of *B. napus* (low erucic acid). A previous study showed that the oil content show no correlation with erucic acid and oleic acid (Smooker et al., 2011), while another research found that oil content showed negative correlation with erucic acid but positive correlation with oleic acid in *B. napus* (Zhao et al., 2008). This resulted indicated that the content of different fatty acid components correlated significantly but varied in different genetic background, especially showing different correlations when the lines contains low erucic acid and high erucic acid.

One of the major objectives of this study was to detect QTL for important agronomic and seed quality traits of *B. carinata*. The QTL we identified here would provide insights for understanding the genetic basis of these traits and further genomic-based genetic improvement of this species, which would be also reference for the comparison with other *Brassica* species. A total of 282 consensus QTL with an average confidence intervals of 6.0 cM, were detected for the investigated traits of the YW DH population, including nine consensus QTL stably detected with major genetic effects for multiple traits (Supplementary Table 5). These major QTL detected stably in different environments may play a key role for the traits being adapted to different environments (Collard et al., 2005). Some QTL identified in the YW DH population were validated in the diverse lines (Table 5). Additionally, new markers associated with the seed quality traits different with that in the YW DH population were also identified. In the YW DH population, the consensus QTL detected in B genome were much more than that in the C genome (Figure 6). This suggested that the allelic variation accounting for the traits in the B subgenome was more than that of the C subgenome between the two parents of the YW DH population. On the other hand, this may also be related to the richer genetic diversity or historical recombination events accumulated in the B genome than that in the C genome of this species, which is indicated by the longer LD decay in the C subgenome (Figure 3).

By physical alignment and comparison of the QTL identified in the YW DH population and diverse lines, we could find homologous QTL accounting for flowering time, erucic acid, and oleic acid traits to the candidate genes and QTL of *B. napus* in the C genome (Figure 8). However, these QTL may have undergone functional differentiation with different genetic effects to the traits and environments between the two species due to different adaptation, domestication and cultivation. For instance, the major QTL for erucic acid in the YW DH population was located in chromosome C4 (identified QTL *qERU39, R*^2^ = 24.53% and *qERU44, R*^2^ = 32.60%, integrated in the consensus *qERU.C4-1*), but the QTL aligned to the major gene in C3 of *B. napus* only presented minor effects in the YW DH population. According to the canola breeding history, the low erucic acid mainly resulted from the mutation of the genes located in A8 and C3 (Harvey and Downey, 1963; Harper et al., 2012). However, in *B. carinata* without the breeding process for low erucic acid such as in the YW DH population, the major QTL was different with that in canola *B. napus*. Therefore, introducing the segment with low erucic acid alleles from the C genome of *B. napus* would be useful for improvement on the seed quality of *B. carinata*. For flowering time, one of the major QTL aligned to the *FT* gene in C6 of *B. napus* could be detected in both of the semi-winter and spring environments in the YW DH population, while this gene may mostly express in spring environment in *B. napus* (Wang et al., 2009). In the near future, with accumulated QTL information from different populations of *B. carianta* and the available genome sequence of this species, we may understand more deeply on the allelic variation within the species and subgenomic variation between the species.

## Author contributions

WZ performed the research, analyzed the data and wrote the manuscript; DH and XS analyzed the data; SG provided the phenotypes in the semi-winter environments; HR and RR performed the experiment in Australia, and analyzed the data; ZW helped the field experiments and phenotyping; JM provided the plant materials and suggestions; JZ designed the research and analyzed the data. All authors revised, read, and approved the final manuscript.

### Conflict of interest statement

The authors declare that the research was conducted in the absence of any commercial or financial relationships that could be construed as a potential conflict of interest. The reviewer EF and handling Editor declared their shared affiliation, and the handling Editor states that the process nevertheless met the standards of a fair and objective review.

## Supplementary material

Supplementary Figure 1 **The meteorology of the different environments investigated during the growing season of ***B. carinata***. (A)** Represents the average temperature each month during the growing season in different environments; **(B)** represents the rainfall each month during the growing season.

Supplementary Figure 2 **The frequency distribution of the phenotypic variation for seven traits of the YW DH population**. Four seed yield related traits and three seed quality traits are showed in this figure, while the other seven investigated traits are shown in Figure 4. P1 and P2 represents the two parents Y-BcDH64 and W-BcDH76, respectively.

Supplementary Figure 3 **Distribution of the consensus QTL accounting for the investigated traits of YW DH population**.

Supplementary Table 1 **Accessions of ***B. carinata*** investigated in this study**.

Supplementary Table 2 **Details of field experiments across multiple environments**.

Supplementary Table 3 **The genetic linkage map of the YW DH population**.

Supplementary Table 4 **The distribution of L(K) and Delta K of population structure analysis**.

Supplementary Table 5 **The identified and consensus QTL for 14 traits investigated in the YW DH population**.

## References

[B1] Altschul S. F. Gish W. Miller W. Myers E. W. Lipman D. J. (1990). Basic local alignment search tool. J. Mol. Evol. 215, 403–410. 2231712 10.1016/S0022-2836(05)80360-2

[B2] Bagheri H. Pino-del-Carpio D. Hanhart C. Bonnema G. Keurentjes J. Aart M. G. M. (2013). Identification of seed-related QTL in *Brassica rapa*. Span. J. Agric. Res. 11, 1085–1093. 10.5424/sjar/2013114-4160

[B3] Bradbury P. J. Zhang Z. Kroon D. E. Casstevens T. M. Ramdoss Y. Buckler E. S. (2007). TASSEL: software for association mapping of complex traits in diverse samples. Bioinformatics 23, 2633–2635. 10.1093/bioinformatics/btm308 17586829

[B4] Chalhoub B. Denoeud F. Liu S. Y. Parkin I. A. P. Tang H. B. Wang X. Y.. (2014). Plant genetics. Early allopolyploid evolution in the post-Neolithic *Brassica napus* oilseed genome. Science 345, 950–953. 10.1126/science.1253435 25146293

[B5] Collard B. C. Y. Jahufer M. Z. Z. Brouwer J. B. Pang E. C. K. (2005). An introduction to markers, quantitative trait loci (QTL) mapping and marker-assisted selection for crop improvement: the basic concepts. Euphytica 142, 169–196. 10.1007/s10681-005-1681-5

[B6] Dhaka N. Rout K. Yadava S. K. Sodhi Y. S. Gupta V. Pental D.. (2016). Genetic dissection of seed weight by QTL analysis and detection of allelic variation in Indian and east European gene pool lines of *Brassica juncea*. Theor. Appl. Genet. 130, 293–307. 10.1007/s00122-016-2811-2 27744489

[B7] Evanno G. Regnaut S. Goudet J. (2005). Detecting the number of clusters of individuals using the software STRUCTURE: a simulation study. Mol. Ecol. 14, 2611–2620. 10.1111/j.1365-294X.2005.02553.x 15969739

[B8] Gan L. Sun X. Jin L. Wang G. Xu J. Wei Z. (2003). Establishment of math models of NIRS analysis for oil and protein contents in seed of *Brassica napus*. Sci. Agric. Sin. 36, 1609–1613.

[B9] Getinet A. Rakow G. Downey R. K. (1996). Aronomic performance and seed quality of Ethiopian mustard in Saskatchewan. Can. J. Plant Sci. 76, 387–392. 10.4141/cjps96-069

[B10] Goffinet B. Gerber S. (2000). Quantitative trait loci: a meta-analysis. Genetics 155, 463–473. 10790417 10.1093/genetics/155.1.463 PMC1461053

[B11] Guo S. M. Zou J. Li R. Y. Long Y. Chen S. Meng J. L. (2012). A genetic linkage map of *Brassica carinata* constructed with a doubled haploid population. Theor. Appl. Genet. 125, 1113–1124. 10.1007/s00122-012-1898-3 22669300

[B12] Harper A. L. Trick M. Higgins J. Fraser F. Clissold L. Wells R.. (2012). Associative transcriptomics of traits in the polyploid crop species *Brassica napus*. Nat. Biotechnol. 30, 798–802. 10.1038/nbt.2302 22820317

[B13] Harvey B. L. Downey R. K. (1963). The inheritance of erucic acid content in rapeseed (*Brassica napus* L). Can. J. Plant Sci. 44, 104–111. 10.4141/cjps64-019

[B14] Hirani A. H. Geng J. F. Zhang J. F. Zelmer C. D. McVetty P. B. E. Daayf F. (2016). Quantitative trait loci mapping and candidate gene identification for seed glucosinolates in *Brassica rapa* L. Crop Sci. 56, 942–956. 10.2135/cropsci2014.12.0837

[B15] Jiang Y. F. Tian E. T. Li R. Y. Chen L. L. Meng J. L. (2007). Genetic diversity of *Brassica carinata* with emphasis on the interspecific crossability with *B. rapa*. Plant Breed. 126, 487–491. 10.1111/j.1439-0523.2007.01393.x

[B16] Körber N. Bus A. Li J. Q. Parkin I. A. P. Wittkop B. Snowdon R. J.. (2016). Agronomic and seed quality traits dissected by genome-wide association mapping in *Brassica napus*. Front. Plant Sci. 7:386. 10.3389/fpls.2016.00386 27066036 PMC4814720

[B17] Li F. Chen B. Y. Xu K. Gao G. Z. Yan G. X. Qiao J. W.. (2016). A genome-wide association study of plant height and primary branch number in rapeseed (*Brassica napus*). Plant Sci. 242, 169–177. 10.1016/j.plantsci.2015.05.012 26566834

[B18] Liu K. Muse S. V. (2005). PowerMarker: an integrated analysis environment for genetic marker analysis. Bioinformatics 21, 2128–2129. 10.1093/bioinformatics/bti282 15705655

[B19] Liu S. Fan C. C. Li J. N. Cai G. Q. Yang Q. Y. Wu J.. (2016). A genome-wide association study reveals novel elite allelic variations in seed oil content of *Brassica napus*. Theor. Appl. Genet. 129, 1203–1215. 10.1007/s00122-016-2697-z 26912143

[B20] Liu S. Y. Liu Y. M. Yang X. H. Tong C. B. Edwards D. Parkin I. A. P.. (2014). The *Brassica oleracea* genome reveals the asymmetrical evolution of polyploid genomes. Nat. Commun. 5:3930. 10.1038/ncomms4930 24852848 PMC4279128

[B21] Lukens L. N. Quijada P. A. Udall J. Pires J. C. Schranz M. E. Osborn T. C. (2004). Genome redundancy and plasticity within ancient and recent *Brassica* crop species. Biol. J. Linn. Soc. 82, 665–674. 10.1111/j.1095-8312.2004.00352.x

[B22] Meng J. L. Shi S. W. Gan L. Li Z. Y. Qu X. S. (1998). The production of yellow-seeded *Brassica napus* (AACC) through crossing interspecific hybrids of *B. campestris* (AA) and *B. carinata* (BBCC) with *B. napus*. Euphytica 103, 329–333. 10.1023/A:1018646223643

[B23] Nei M. Tajima F. Tateno Y. (1983). Accuracy of estimated phylogenetic trees from molecular data II. Gene frequency data. J. Mol. Evol. 19, 153–170. 10.1007/BF02300753 6571220

[B24] Parkin I. A. P. Koh C. Tang H. B. Robinson S. J. Kagale S. Clarke W. E.. (2014). Transcriptome and methylome profiling reveals relics of genome dominance in the mesopolyploid *Brassica oleracea*. Genome Biol. 15:R77. 10.1186/gb-2014-15-6-r77 24916971 PMC4097860

[B25] Pelc S. E. Couillard D. M. Stansell Z. J. Farnham M. W. (2015). Genetic diversity and population structure of collard landraces and their relationship to other *Brassica oleracea* Crops. Plant Genome 8, 1–11. 10.3835/plantgenome2015.04.0023 33228266

[B26] Pritchard J. K. Stephens M. Donnelly P. (2000). Inference of population structure using multilocus genotype data. Genetics 155, 945–959. 10835412 10.1093/genetics/155.2.945 PMC1461096

[B27] Qian L. W. Qian W. Snowdon R. J. (2014). Sub-genomic selection patterns as a signature of breeding in the allopolyploid *Brassica napus* genome. BMC Genomics 15:1170. 10.1186/1471-2164-15-1170 25539568 PMC4367848

[B28] Rahman M. Mamidi S. McClean P. (2014). Quantitative trait loci mapping of seed colour, hairy leaf, seedling anthocyanin, leaf chlorosis and days to flowering in F2 population of *Brassica rapa* L. Plant Breed. 133, 381–389. 10.1111/pbr.12165

[B29] Raman H. Raman R. Coombes N. Song J. Diffey S. Kilian A.. (2016). Genome-wide association study identifies new loci for resistance to *Leptosphaeria maculans* in canola. Front. Plant Sci. 7:1513. 10.3389/fpls.2016.01513 27822217 PMC5075532

[B30] Raman H. Raman R. Kilian A. Detering F. Carling J. Coombes N.. (2014). Genome-wide delineation of natural variation for pod shatter resistance in *Brassica napus*. PLoS ONE 9:e101673. 10.1371/journal.pone.0101673 25006804 PMC4090071

[B31] Remington D. L. Thornsberry J. M. Matsuoka Y. Wilson L. M. Whitt S. R. Doebley J.. (2001). Structure of linkage disequilibrium and phenotypic associations in the maize genome. Proc. Natl. Acad. Sci. U.S.A. 98, 11479–11484. 10.1073/pnas.201394398 11562485 PMC58755

[B32] SAS Institute Inc (2000). SAS/STAT User's Guide, Version 8. Cary, NC: SAS Institute Inc.

[B33] Shi J. Q. Li R. Y. Qiu D. Jiang C. C. Long Y. Morgan C.. (2009). Unraveling the complex trait of crop yield with quantitative trait loci mapping in *Brassica napus*. Genetics 182, 851–861. 10.1534/genetics.109.101642 19414564 PMC2710164

[B34] Singh S. Mohapatra T. Singh N. Hussain Z. (2012). Mapping of QTLs for oil content and fatty acid composition in Indian mustard [*Brassica juncea* (L.) Czern. and Coss.]. J. Plant Biochem. Biotechnol. 22, 80–89. 10.1007/s13562-012-0113-6

[B35] Smooker A. M. Wells S. Morgan C. Beaudoin F. Cho K. Fraser F.. (2011). The identification and mapping of candidate genes and QTL involved in the fatty acid desaturation pathway in *Brassica napus*. Theor. Appl. Genet. 122, 1075–1090. 10.1007/s00122-010-1512-5 21184048

[B36] Snowdon R. J. Friedt W. (2004). Molecular markers in *Brassica* oilseed breeding: current status and future possibilities. Plant Breedi. 123, 1–8. 10.1111/j.1439-0523.2003.00968.x

[B37] Tamura K. Dudley J. Nei M. Kumar S. (2007). MEGA4: Molecular Evolutionary Genetics Analysis (MEGA) software version 4.0. Mol. Biol. Evol. 24, 1596–1599. 10.1093/molbev/msm092 17488738

[B38] Nagaharu N. (1935). Genomic analysis in *Brassica* with special reference to the experimental formation of *B. napus* and peculiar mode of fertilization. Jpn. J. Bot. 7, 389–452.

[B39] Wang J. Long Y. Wu B. D. Liu J. Jiang C. C. Shi J. Q.. (2009). The evolution of *Brassica napus FLOWERING LOCUST* paralogues in the context of inverted chromosomal duplication blocks. BMC Evol. Biol. 9:271. 10.1186/1471-2148-9-271 19939256 PMC2794288

[B40] Wang S. C. Basten C. J. Zeng Z.-B. (2012). Windows QTL Cartographer 2.5. Department of Statistics, North Carolina State University, Raleigh, NC.

[B41] Wang X. W. Wang H. Z. Wang J. Sun R. F. Wu J. Liu S. Y.. (2011). The genome of the mesopolyploid crop species *Brassica rapa*. Nat. Genet. 43, 1035–1157. 10.1038/ng.919 21873998

[B42] Warwick S. I. (2011). Brassicaceae in agriculture, in Genetics and Genomics of the Brassicaceae, eds Schmidt R. Bancroft I. (New York, NY: Springer Science + Business Media; LLC), 33–65.

[B43] Warwick S. I. Gugel R. K. McDonald T. Falk K. C. (2006). Genetic variation of Ethiopian mustard (*Brassica carinata* A. Braun) germplasm in western Canada. Genet. Resour. Crop Evol. 53, 297–312. 10.1007/s10722-004-6108-y

[B44] Weaver B. Wuensch K. L. (2013). SPSS and SAS programs for comparing Pearson correlations and OLS regression coefficients. Behav. Res. Methods 45, 880–895. 10.3758/s13428-012-0289-7 23344734

[B45] Wei Z. L. Wang M. Chang S. H. Wu C. Liu P. F. Meng J. L.. (2016). Introgressing subgenome components from *Brassica rapa* and *B. carinata* to *B. juncea* for broadening its genetic base and exploring intersubgenomic heterosis. Front. Plant Sci. 7:1677. 10.3389/fpls.2016.01677 27909440 PMC5112257

[B46] Xiao Y. Chen L. L. Zou J. Tian E. T. Xia W. Meng J. L. (2010). Development of a population for substantial new type *Brassica napus* diversified at both A/C genomes. Theor. Appl. Genet. 121, 1141–1150. 10.1007/s00122-010-1378-6 20556596

[B47] Yang J. H. Liu D. Y. Wang X. W. Ji C. M. Cheng F. Liu B. N.. (2016). The genome sequence of allopolyploid *Brassica juncea* and analysis of differential homoeolog gene expression influencing selection. Nat. Genet. 48, 1225–1232. 10.1038/ng.3657 27595476

[B48] Yong H. Y. Wang C. L. Bancroft I. Li F. Wu X. M. Kitashiba H.. (2015). Identification of a gene controlling variation in the salt tolerance of rapeseed (*Brassica napus* L.). Planta 242, 313–326. 10.1007/s00425-015-2310-8 25921693

[B49] Zhao J. Y. Dimov Z. Becker H. C. Ecke W. G. Mollers C. (2008). Mapping QTL controlling fatty acid composition in a doubled haploid rapeseed population segregating for oil content. Mol. Breed. 21, 115–125. 10.1007/s11032-007-9113-y

[B50] Zou J. Jiang C. C. Cao Z. Y. Li R. Y. Long Y. Chen S.. (2010). Association mapping of seed oil content in different *Brassica napus* populations and its coincidence with QTL identified from linkage mapping. Genome 53, 908–916. 10.1139/G10-075 21076506

[B51] Zou J. Raman H. Guo S. M. Hu D. D. Wei Z. L. Luo Z. L.. (2014). Constructing a dense genetic linkage map and mapping QTL for the traits of flower development in *Brassica carinata*. Theor. Appl. Genet. 127, 1593–1605. 10.1007/s00122-014-2321-z 24824567

