# Supplementary figures and images for: Investigation of the Genetic Diversity and Quantitative Trait Loci Accounting for Important Agronomic and Seed Quality Traits in Brassica carinata
Source: Front Plant Sci. 2017 Apr 24;8:615. doi: 10.3389/fpls.2017.00615 (PMC5401912; doi:10.3389/fpls.2017.00615)

A

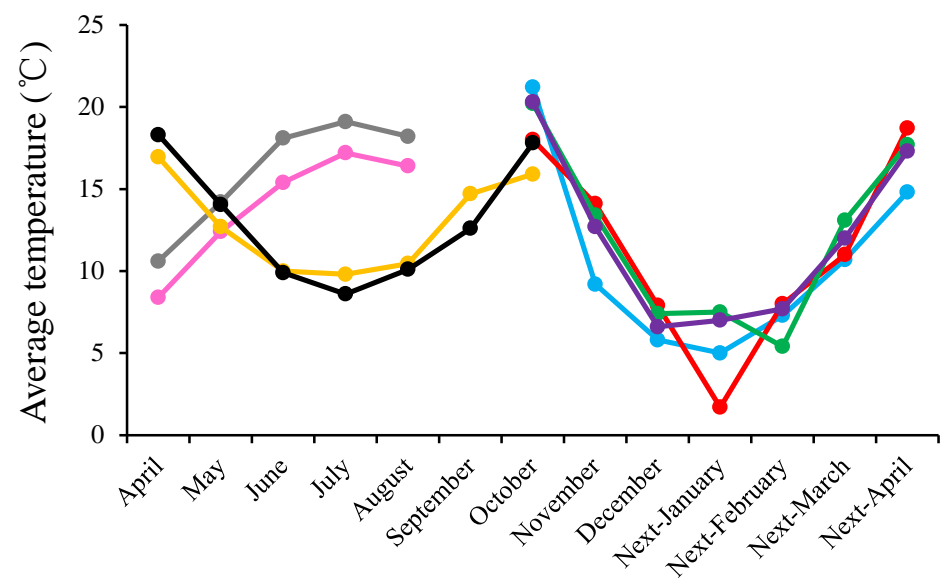

09WH 10WH  
13WH 14WH

B

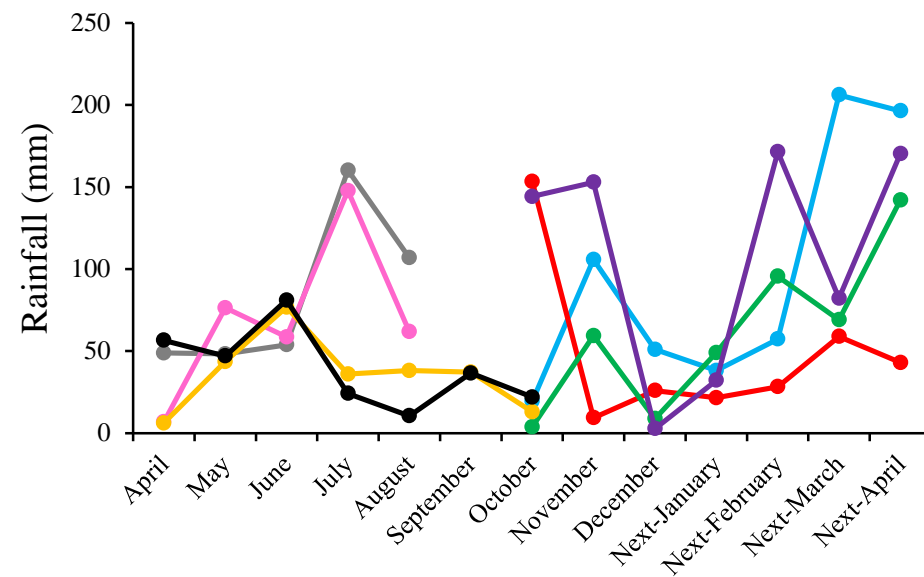

12HZ 12XN  
13WW 14WW

Supplement: Supplementary Figure 1 — The meteorology of the different environments investigated during the growing season of B. carinata. (A) Represents the average temperature each month during the growing season in different environments; (B) represents the rainfall each month during the growing season. [file Image1.PDF]

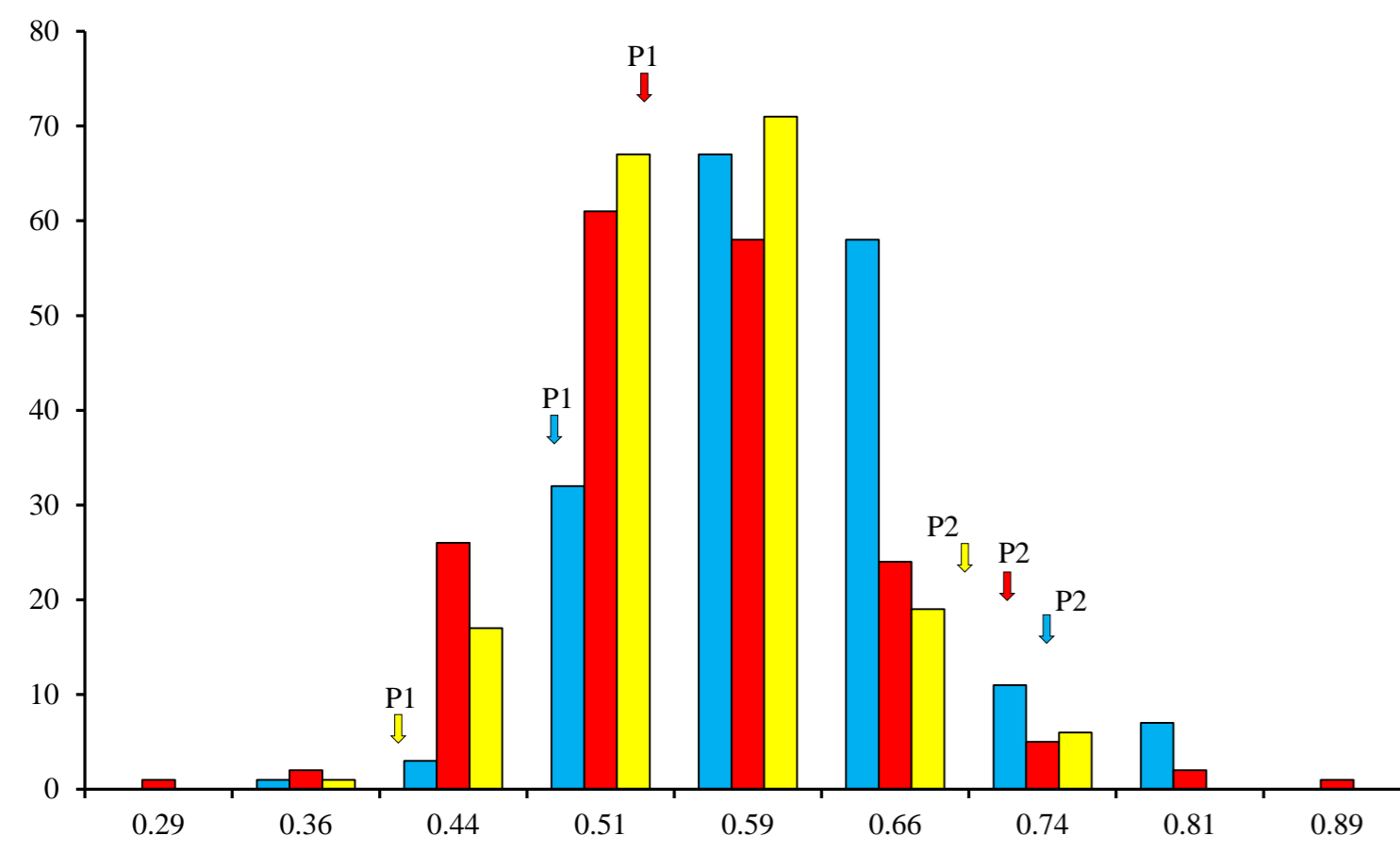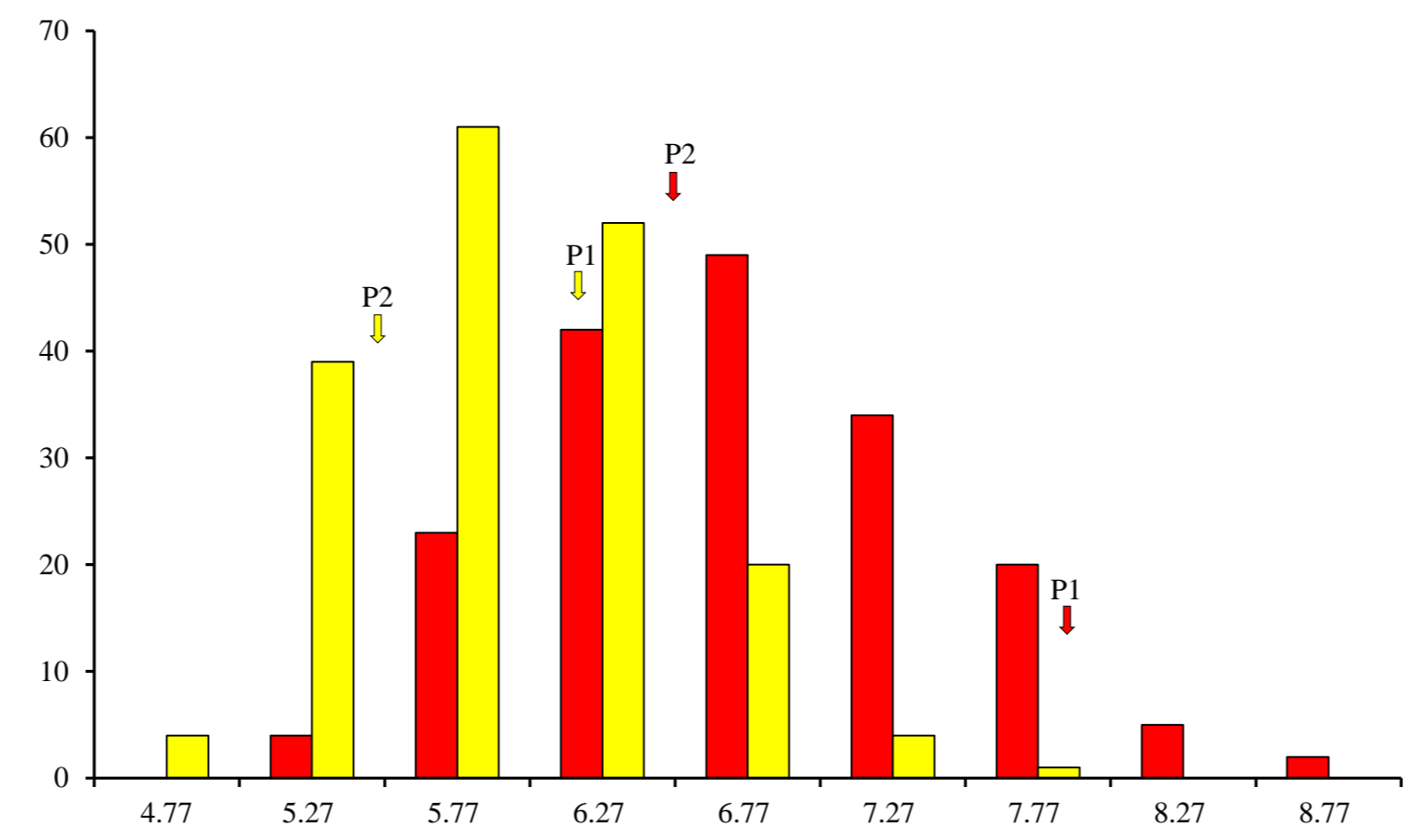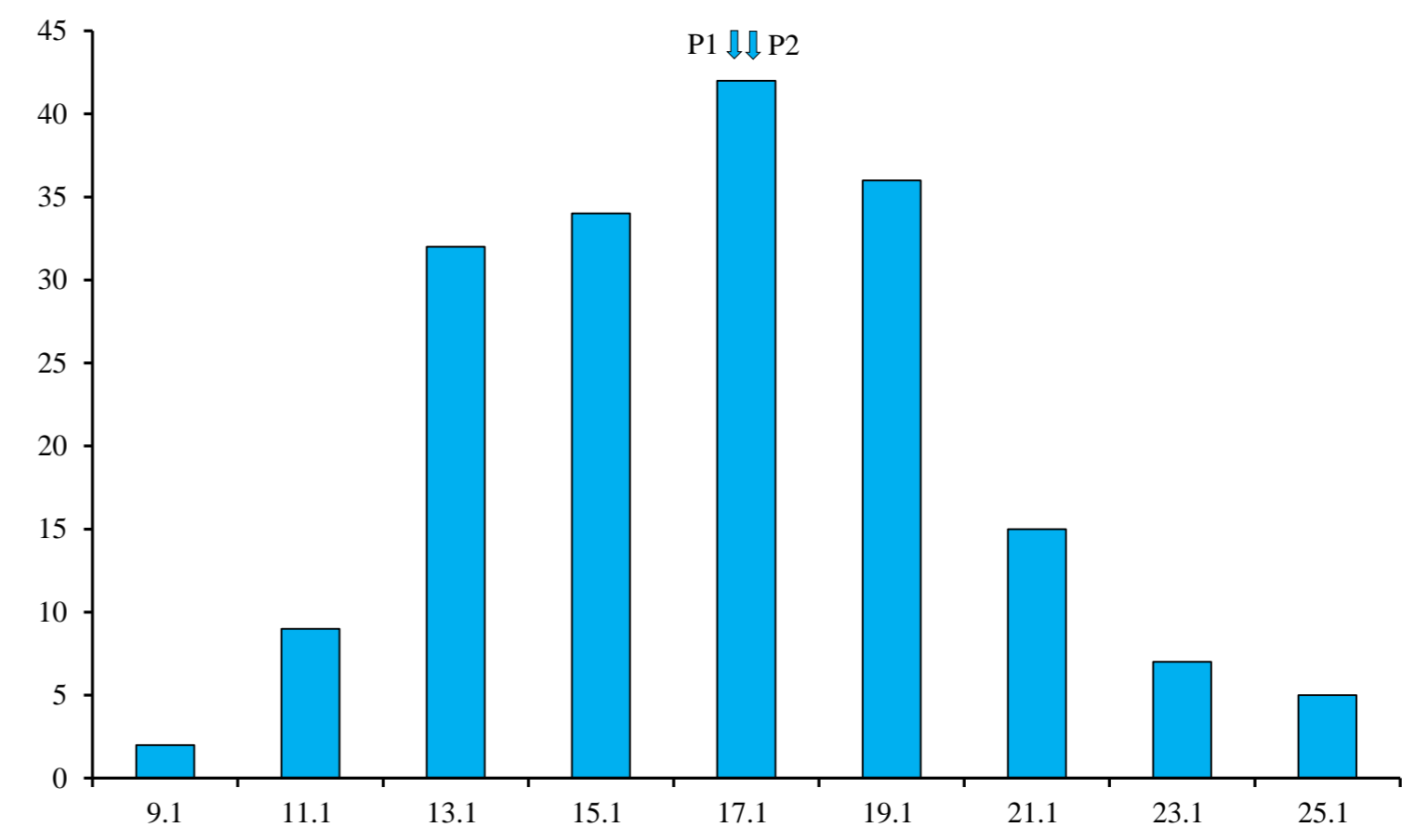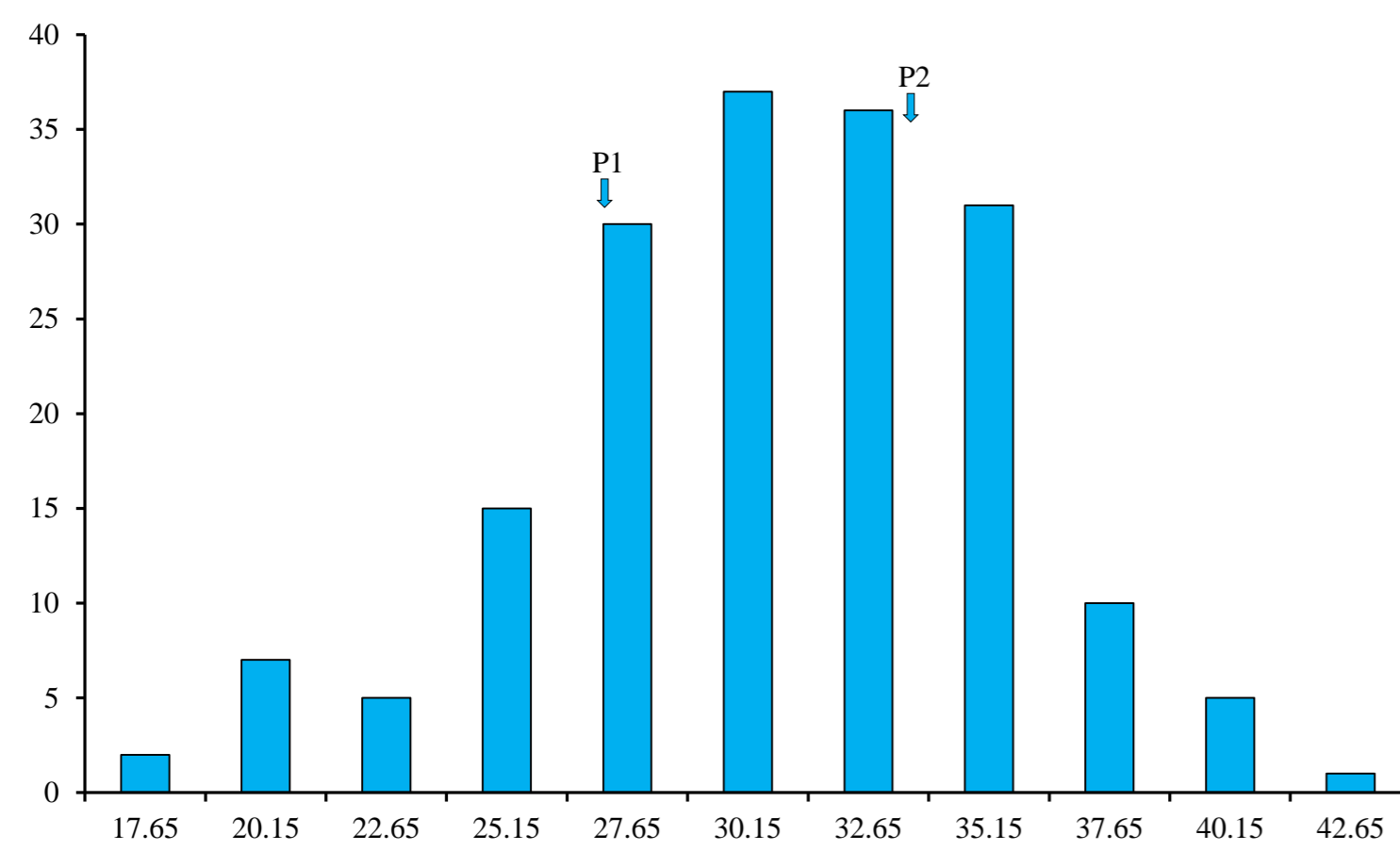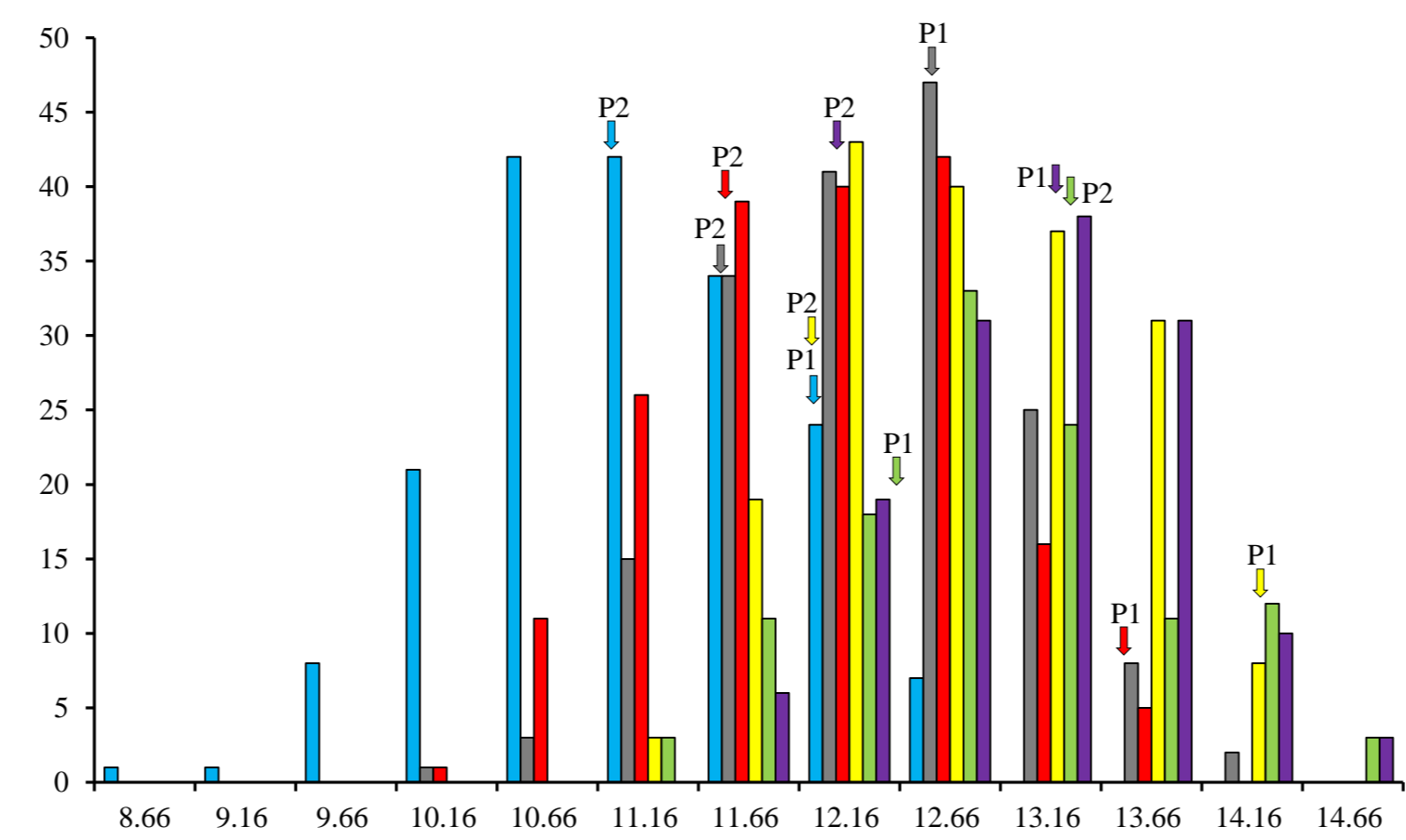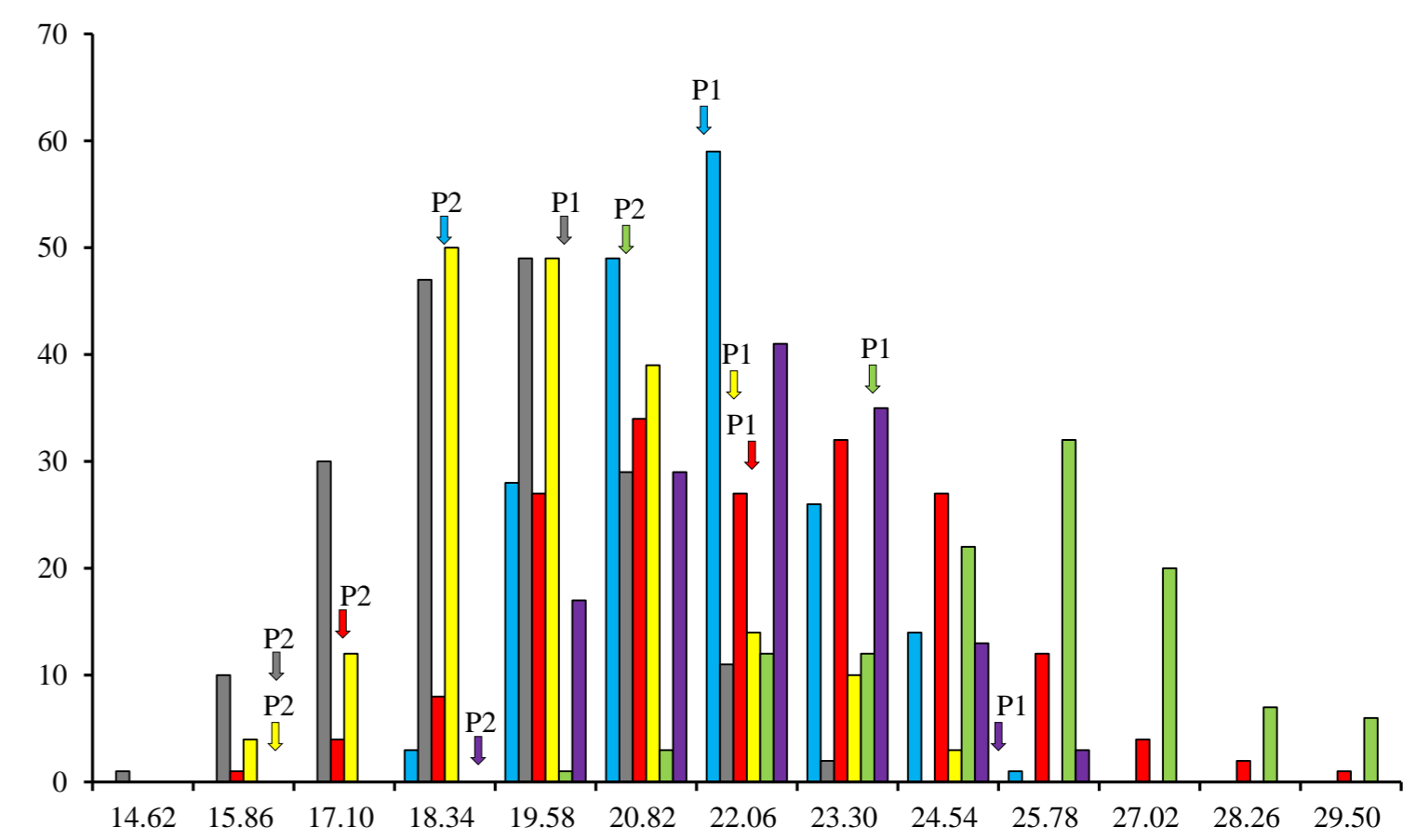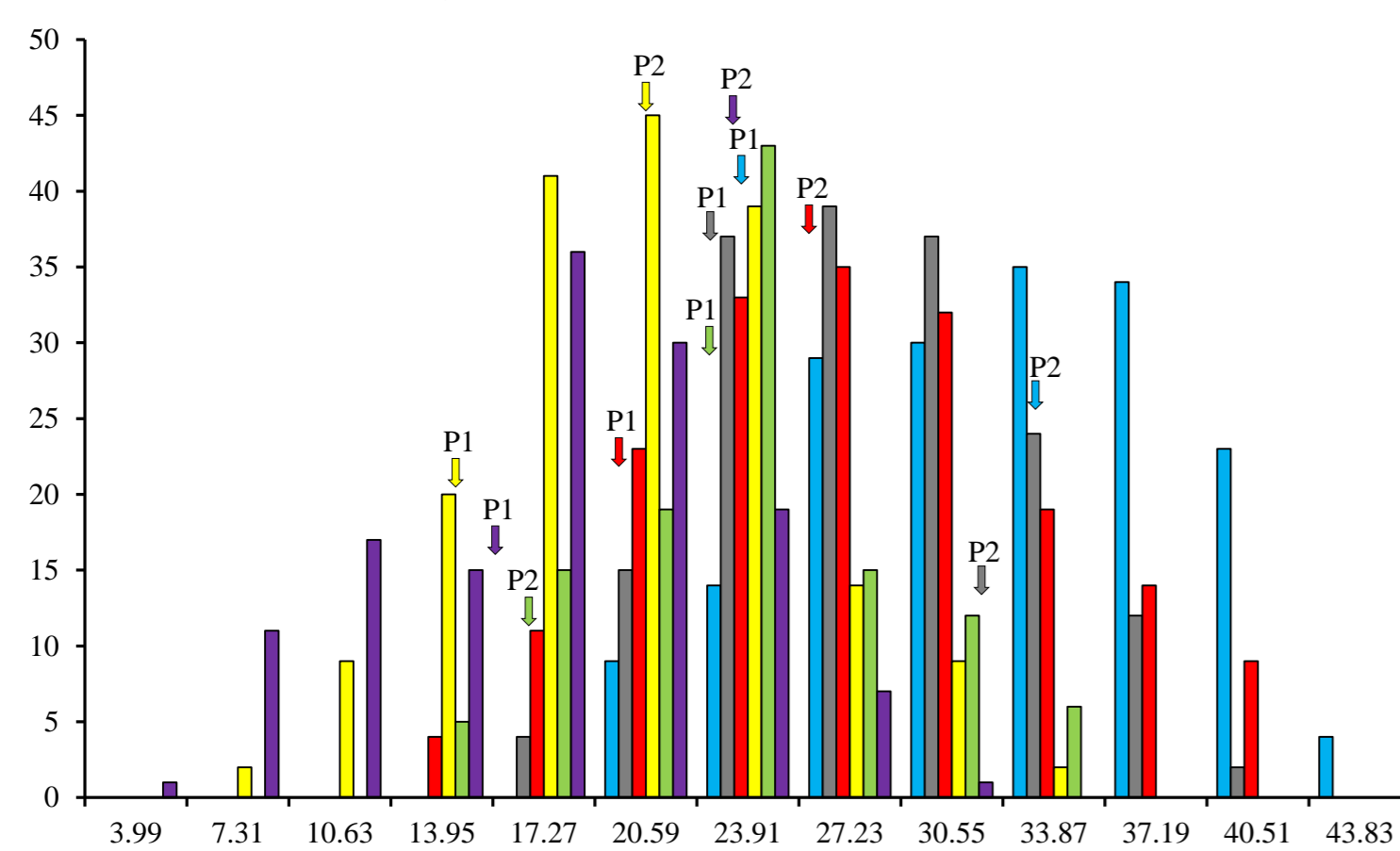

- WH09
- WH10
- HZ12
- XN12
- WH13
- WH14

Supplement: Supplementary Figure 2 — The frequency distribution of the phenotypic variation for seven traits of the YW DH population. Four seed yield related traits and three seed quality traits are showed in this figure, while the other seven investigated traits are shown in Figure 4. P1 and P2 represents the two parents Y-BcDH64 and W-BcDH76, respectively. [file Image2.PDF]
