# Supplementary material for: Investigation of the Genetic Diversity and Quantitative Trait Loci Accounting for Important Agronomic and Seed Quality Traits in Brassica carinata
Source: Front Plant Sci. 2017 Apr 24;8:615. doi: 10.3389/fpls.2017.00615 (PMC5401912; doi:10.3389/fpls.2017.00615)
Supplement: Supplementary Figure 3 — Distribution of the consensus QTL accounting for the investigated traits of YW DH population. [file Image3.PDF]

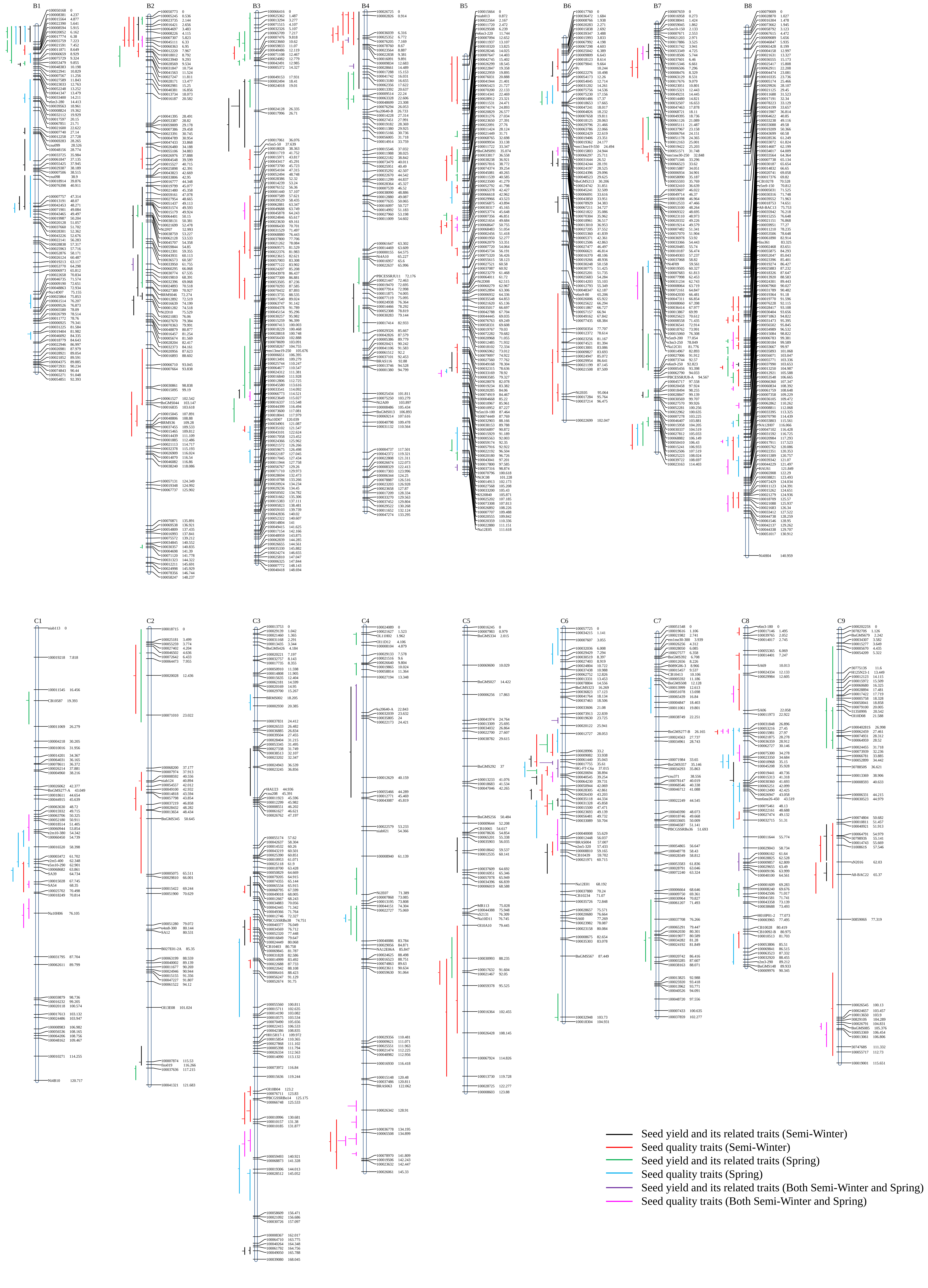
B1
B2
B3
B4
B5
B6
B7
B8
C1
C2
C3
C4
C5
C6
C7
C8
C9
Seed yield and its related traits (Semi-Winter)
Seed quality traits (Semi-Winter)
Seed yield and its related traits (Spring)
Seed quality traits (Spring)
Seed yield and its related traits (Both Semi-Winter and Spring)
Seed quality traits (Both Semi-Winter and Spring)
